# Cross-sectional study of height and weight in the population of Andalusia from age 3 to adulthood

**DOI:** 10.1186/1472-6823-8-S1-S1

**Published:** 2008-07-18

**Authors:** Juan Pedro López-Siguero, Juan Manuel Fernández García, Juan de Dios Luna Castillo, Jose Antonio Moreno Molina, Carlos Ruiz Cosano, Antonio Jurado Ortiz

**Affiliations:** 1 Paediatric Endocrinology Department, Hospital Materno Infantil, Malaga, Spain; 2 Paediatrics Department, University Clinical Hospital of Granada, Granada, Spain; 3 Faculty of Medicine, University of Granada, Granada, Spain; 4 "El Limonar" Health Centre, Malaga, Malaga, Spain; 5 Paediatrics Department, Regional University Hospital of Malaga, Malaga, Spain

## Abstract

**Background and objectives:**

In Andalusia there were no studies including a representative sample of children and adolescent population assessing growth and weight increase. Our objectives were to develop reference standards for weight, height and BMI for the Andalusian pediatric population, from 3 to 18 years of age for both genders, and to identify the final adult height in Andalusia.

**Subjects and methods:**

Two samples were collected. The first included individuals from 3 to 18 years of age (3592 girls and 3605 boys). They were stratified according type of study center, size of population of origin, age (32 categories of 0.5 years) and gender, using cluster sampling. Subjects from >18 to 23 years of age (947 women and 921 men) were sampled in 6 non-university educational centers and several university centers in Granada. Exclusion criteria included sons of non-Spanish mother or father, and individuals with chronic conditions and/or therapies affecting growth. Two trained fellows collected the data through February to December 2004, for the first sample, and through January to May 2005, for the second.

Reference curves were adjusted using Cole's LMS method, and the quality of the adjustment was assessed using the tests proposed by Royston. In addition, a sensitivity analysis was applied to the final models obtained.

**Results:**

Data for 9065 cases (4539 women and 4526 men) were obtained; 79.39% (n = 7197) in the up to 18 years of age group. In the first sampling only 0.07% (3 girls and 2 boys) refused to participate in the study. In addition, 327 students (4.5%) were absent when sampling was done. We present mean and standard deviation fort height, weight and BMI at 0.5 years intervals, from 3 to 23 years of age, for both genders. After adjustment with the different models, percentiles for height, weight (percentiles 3, 5, 10, 25, 50, 75, 90, 95, and 97) and BMI (percentiles 3, 5, 50, 85, 95, and 97) are presented for both genders.

**Conclusion:**

This is the first study in Andalusia with a representative sample from the child-juvenile population to investigate weight, height and BMI in subjects from 3 to 23 years of age. The great variability observed in the values from sample of 18 to 23 years of age individuals, ensures the inclusion of extreme values, although random sampling was not used. There still is a lack of standard reference values for the Andalusian population younger done 3 years of age.

## Introduction

Growth is a complex biological process whereby an organism achieves an increase in mass and size, while at the same time it matures morphologically and functionally until it acquires the characteristics of the adult state. It is a process that is genetically determined by the activation of stimulating and inhibiting genes, but modulated by extragenetic factors, so that the rhythm of maturing and the final size are the result of a complex interaction between genes and environment.

Growth is the fundamental physiological process that characterizes childhood. It should be monitored by a paediatrician and the family and considered a health indicator. In a similar way, secular trends in growth show the level of health of the population itself.

References of growth are one of the most valuable and commonly used instruments in the evaluation of the well-being of individuals, groups of children and the communities they live in, and for following this process in achieving a series of sanitary and other wider targets related to social equality. This is due to the well-known fact that the improvement in socio-sanitary and nutritional conditions leads to growth acceleration in a determined population.

Auxological anthropometry is a combination of biometric techniques applied to the study and evaluation of growth. The use of this tool brings a series of data about the population (weight, height, perimeters, etc) and creates a model which can be used as a standard for this population, if it is representative of them.

A correct assessment of a growth pattern requires the comparison of the subject's data with standards obtained from a representative sample of the population the subject belongs to. Such standards can be elaborated by using three methods, which differ in the way the sample is chosen and followed: the transversal, the longitudinal and the semi-longitudinal method.

Studies with a transversal design for the creation of weight and height tables carry out a single resolution to determine these parameters in a sample that represents the population it proceeds from. Longitudinal studies follow the sample through its process of growth and development. When the data obtained from longitudinal and transversal studies are adequately compared, they are found to be virtually interchangeable beyond the pubertal growth spurt. In transversal studies, it is not possible to create growth rate tables or to adequately monitor growth during puberty.

From the moment of their publication in 1965, Tanner and Whitehouse's tables [1], which were later updated [2], have been widely used for the evaluation of weight and height of populations even outside Great Britain. In our country, these tables have been used for a long time in clinical practice for the anthropometric evaluation in our patients. Currently, they are still used in hospitals and primary healthcare centres, even though it has been proven that they are now obsolete and do not represent population nowadays [3]. In the United Kingdom, where these tables are no longer used, there has been widespread confusion about which tables are most appropriate for clinical use. A working group at the request of the Royal College of Paediatrics and Child Health, has assessed the available tables and recommended the tables known as UK 90 [4] for clinical use. These have since been validated as a tool for monitoring growth in the British population [5].

A broad study which took place in 10 European countries, including Spain, showed that there are still international differences in the growth of the child population [6]. For this reason, the scientific community recommends the use of local height and weight standards in the clinical assessment of patients.

On the other hand, we live in a complex and multiracial society. It is difficult or even impossible to carry out an auxological evaluation of an immigrant child in our country, due to the lack of standards from their country of origin. In these circumstances, graphs from the adopted country are used, given that environmental factors are fundamental when the child receives adequate nutrition and care from birth [7], while posterior improvement in environmental conditions optimises the genetic load expressiveness. Another option is the use of the WHO graphs. The Multicentre Growth Reference Study (MGRS) aims to generate growth references for infants and children until 5 years of age. The sample was made up of 8,500 children from 6 countries (Brazil, Ghana, India, Norway, Oman and the US). The 60-month study demonstrated that these standards can be used to assess the growth of any child regardless their race, socio-economic level or nutrition so long as optimum environmental conditions are met [8].

In Spain, no home-grown, widely diffused standards were developed, until the Bilbao study that was carried out from 1982 to 1988 [9]. This study used a mixed, semi-longitudinal design with three groups of 600 children who were followed for 9 years in the population of greater Bilbao with middle to low socio-economic level. Data were published for subjects from 0 to 18 years. The same group published the end of its longitudinal study in 2004 (children born between 1978 and 1980) and the transversal study carried out between 2000 and 2001, with a sample of 6443 subjects between 0 and 18 years old [10].

In the Community of Murcia, another transversal study of the child population aged between 4 and 17 (1,930 children) was undertaken [11] while in Madrid a study was carried out with 1,525 schoolchildren between the ages of 6 and 18 [12].

During the same period (2005), an important longitudinal study was published in Aragon on a final sample (of adult size) of 226 subjects [13]. This study is of great importance because it considers diverse anthropometric measures and intellectual development, with the possibility of calculating growth rate in all ages, including puberty, which enables the assessment of subjects with different pubescent "tempos". To date, no transversal study is available for this population.

In 2004, the Barcelona transversal study was published [14], which included newborns, infants, children and adolescents, as well as young adults for the assessment of adult size. The sample of 3- to 18-year olds is made up of 5,257 children and adolescents measured in 2002 and 2003. The study concludes with the demonstration of a secular growth of 3.5 cm in respect to a previous Catalan study carried out in 1985 [15] and the recommendation of periodically updating auxological data.

Weight and body mass index (BMI), weight quotient (kg)/height^2^(m^2^), are two widely used parameters for the assessment of nutritional state since they are related to the total amount of body fat. Obesity is becoming a public health problem in our country and statistics warn us that in Andalusia this is going to be a challenge for health professionals dedicated to children's healthcare [16,17]. The EnKid [18] study on the prevalence of obesity in Spain among 2- to 24-year olds has shown a prevalence of obesity in our country of 13.9% and 26.3% if we also consider those who are overweight. Moreover, in this study, Andalusia and the Canaries are the communities with higher prevalence (29.4% ponderal excess in the case of Andalusia). This study was carried out between 1998 and 2000, with a sample of 3,534 subjects.

Data connecting childhood and adolescent obesity with metabolism disorders in adulthood make it necessary to set up studies to find out about the reality of the situation in Andalusia. Such knowledge should be the starting point for the development of intervention programmes to control this emerging problem.

In Andalusia, there was no representative study of the child and adolescent population. Given the scientific community's recommendation of using local standards to assess growth and weight increase in our population, it was decided to initiate a study with the objective of obtaining height and weight tables that can be used as a reference for our population. This would be the first study of growth in our community that would create height and weight tables representative and usable in daily clinical practice. It would also enable us to find out the final adult height of the Andalusian population.

The objective of the work was to describe the growth in height and weight of the child and youth population of Andalusia from the age of 3 to 18, including both sexes, and to create reference standards for weight, height and BMI for that part of the Andalusian population that would be useful in daily clinical practice and to discover the final adult height of the Andalusian population.

## Subjects and methodology

### Population studied and sample selected

#### Target population and considerations about the sample

Once the objectives were clear, it was necessary to make decisions about the sample that would be the object of our study, bearing in mind that a transversal study had been chosen.

In order to achieve the greatest possible representation of the Andalusian population from the age of 3 until they reach final height two samples of individuals were obtained using different sampling systems to assess their weight and height.

Given that the aim of the first sample was to describe the growth in height and weight of the Andalusian child and youth population from the age of 3 to 18 including both sexes and create standards of reference for weight, height and BMI for the Andalusian population within this age range, this first sample needed to represent the whole Andalusian population from the age of 3 to 18. The ideal method, from a methodological viewpoint, for taking a sample of that population would be through simple random sampling taken from the population census; however, this would not be viable, economically speaking. We could obtain a less expensive sample by going to places that are attended by children of these ages, to weigh and measure them.

These places are, of course, education centres, where the population between the ages of 3 and 16 is obliged to attend. Moreover, apart from the age range, if the schooling rate is high enough, a fairly reasonable degree of representation could be achieved.

Data from the Education, Science, and Sports Ministry, show that in 2002–2003 the schooling rate in Andalusia was 96.1% for High school and Professional Education (16–17 years of age) and 17.4% for higher grades of Professional Education (18–19 years of age). Thus, up to the age of 17, the schooling rate in Andalusia is high enough to avoid significant skewness when it comes to taking samples of children and young people from education centres. At the age of 18, the number of students in non-university education is very low and makes it very difficult to sample this age group using only non-university education institutions.

On the other hand, the data from the 2001 census by the National Statistics Institute (Instituto Nacional de Estadística) about rates of schooling (at any level, including university) indicate that, at that time, the schooling rate for 16-year olds was 81.9%, for 17-year olds 71.8% and for 18-year olds 62.2%. Therefore, population aged 16 and 17 would be sufficiently covered by going to education centres, while for 18-year olds it would be necessary to obtain data from other sources in order to cover such deficiencies.

Despite everything, the problem was not serious given that the age where we might encounter problems is one where the variance is very large. This variance is present in all population strata so that the results obtained would not be far from reality in any case.

It was decided to take a random sample per school and within such sample select classes from each year, from which a sample of pupils in the age range being considered would be taken. This sample (despite, as explained later, the risk being reduced to a minimum) could introduce skewness in the assessment due to the resemblance of the pupils in each class. This skewness could artificially reduce the variance of the parameter studied at a certain age, thus strongly affecting the assessment of extreme percentiles. It will be corrected by using adjustments with random effects models to take this fact into account.

For the second sample, concerning the young population between the ages of 18 and 23, it was not viable to use the same sampling method. For this group, 6 non-university education centres were used, where different professional modules were taught and where data were collected from classes in a similar way to the sample of under 18-year olds. In addition, to increase the size of the sample, different university departments in Granada were selected: The School of Health Sciences, The Medicine Faculty, The Civil Engineering School, The Pharmacy Faculty, The Science Faculty and the Computer Engineering School. In all these centres, students in their first, second and third years were measured.

Given the economic determinants, although the statistical quality of this second sample was worse due to the fact that it was not random, this type of sampling was chosen assuming that the strong variance of the different measures would assure a representation of extreme values that would not be reduced even though the sample was not random. On the other hand, if, as was expected, from the age of 18 or 19 the behaviour of the measurements was asyntotic, ages could be put in one group where extreme measures are more probable thus correcting a possible skewness, stemming from underestimating the population's variance.

#### Types of education centres and sample size

According to data from the Andalusian Board of Education for the academic year 2003–2004, education centres were classified as Private Teaching Centres, Infant and Primary Schools and Institutes for Secondary Education. Moreover, centres are not evenly distributed across the region, but rather they are placed according to the size of the population in a given area. Therefore, there were two already identified criteria to stratify: the type of centre and the size of the population.

Table 1 shows data about the types of centres, the size of the population and the number of pupils.

**Table 1:** Distribution of Education Centres and pupils by strata

**Population in the town of origin**	**Type of Education Centre**	**No. of Centres**	**Centres (%)**	**No. of students**	**Students (%)**
≥ 100,000	Private education centres	361	11.5	218778	16.0
	Infant and primary schools	430	13.7	149631	10.9
	Secondary school	247	7.8	156305	11.4
50,000–99,999	Private education centres	55	1.7	29296	2.1
	Infant and primary schools	147	4.7	56954	4.2
	Secondary school	66	2.1	46152	3.4
20,000–49,999	Private education centres	54	1.7	27738	2.0
	Infant and primary schools	145	4.6	57361	4.2
	Secondary school	69	2.2	45390	3.3
<20,000	Private education centres	111	3.5	43360	3.2
	Infant and primary schools	1002	31.8	333087	24.4
	Secondary school	461	14.6	202872	14.8
		3148		1366924	

The global sample was distributed into 12 strata, in principle, in proportion to the number of pupils in each stratum. Although knowing the distribution of the population in the centres and the pupils in each stratum, it is always possible to calculate the probability of one unit being selected for sampling, and correct this using inverse probability weighting, in case an imbalance in the sample by strata is considered necessary. As explained below, this was the strategy used for sampling.

Considering our objective, an additional source for stratification would be each of the age groups from 3 to 18, which would give us 16 strata. In fact, for the sake of accuracy, we would consider not 16 strata, but 32, given that we would choose children of an age calculated in years and "half years"; this way strata would reflect the following ages: 3, 3.5, 4, 4.5, 5, 5.5, .....17, 17.5, 18, 18.5.

Besides, another obvious source of stratification would be the child's sex, due to the fact that the variables measured are different from a very early age in boys and girls.

In terms of the calculation of the sample size we should comment on the normality of the variables involved in the study. For each age, height can be considered to follow a normal distribution, but this is not the case for weight or BMI, calculated based on both of these. Despite this, many authors have managed to normalize these variables using transformations; this way, we consider that if the children's weight for each age group is not a normal variable, once transformed it would become normal, which means that we would not have to re-calculate using non-parametric methods for the size of the sample.

Having discussed the prior considerations, we can concentrate on the calculation of the sample size necessary for the study. Following the methodology set out by Linnet [19] for the calculation of sample size, the key would be the ratio between the width of reference range and the width of the confidence interval for the extremes of the said reference range. Accordingly, for a reasonable quotient between both widths, such as 20%, we would need 126 children per each age group and sex.

However, these calculations do not take into account that the percentiles in the height and weight curves for age are obtained from the ratio of such variables with age using a regression model. Royston [20] addresses this problem, and using his equations the required sample size is reduced due to the variance reduction that occurs when using regression. In figures, such reduction means that if we want a ratio of 0.20 for each group of age and sex, we would only need 74 children. Based on this fact and bearing in mind that it would be applicable to simple random sampling, the size of our global sample would be at least 2,368 males and 2,368 females.

We had opted for a multistage sample. The first stage included a random selection of the education centre, while the second stage included a random selection of the children within the class of their year in the education centre. This leads to a cluster sampling that requires an increase in the size of the sample. Such increase, which is called the design effect, depends on the size of the sample in each centre as well as the degree of resemblance between children in each centre (intraclass correlation coefficient). The larger the effect of the intraclass coefficient, and the larger the sample group in each centre, the larger the design effect. This design effect shows us a coefficient by which we should multiply the size of the sample calculated as a simple random sample so that when we obtain it in clusters we have the same capacity as a simple random sample.

We would choose our sample for each age group (3, 3.5, 4, 4.5 years...) so that in fact our sample would not have to be too big; it would normally be about 4 or 5 pupils in each age group per school. So, supposing we chose 4 children of each age from all the children of the school and that the intraclass correlation coefficient was 5%, the value of the design effect would be 1.15. Therefore, our total sample would be approximately 1.15 × 4608 ≈ 5300 pupils of whom half would be female and half male. This means around 166 pupils per age group of whom half would be female and half male.

The choice of 0.05 as the intra-class correlation coefficient was made on the assumption that although the children in class would resemble each other, variance increases with age. Therefore, in classes of older children variance between observations is guaranteed, while in the case of younger children an important variance is also guaranteed due to the fact that the age range within a class, although small, is strong enough to mean that the intra-class correlation is not very large.

In the case of the sample of students between the ages of 18 and 23, the size of the sample calculated following the same steps included 2000 people.

#### Sample distribution in education centres

Taking into account the sizes of the samples obtained, the global sample would be distributed in the 12 strata, in principle, in proportion to the number of pupils in each stratum, as we knew the distribution of centres by strata and their mean size. This allocation can be seen in the column "Proportional sample" in Table 2.

**Table 2:** Sample distribution in Education Centres by Strata

**Population in the town of origin**	**Type of Education Centre**	**Proportional sample**	**Definite sample**
≥ 100,000	Private education centres	13	15
	Infant and primary schools	9	19
	Secondary school	10	16
50,000–99,999	Private education centres	2	2
	Infant and primary schools	3	3
	Secondary school	3	3
20,000–49,999	Private education centres	2	2
	Infant and primary schools	4	4
	Secondary school	3	3
<20,000	Private education centres	3	2
	Infant and primary schools	20	10
	Secondary school	12	6
		84	85

As discussed earlier, knowing the distribution of the population in the centres and that of pupils in each stratum, it is always possible to calculate the probability of a unit being selected for sampling and to correct using inverse probability weighting, if an imbalance in the sample strata is considered necessary.

We made use of this methodological resource, with the exact intention of under-representing samples from centres with a smaller population, since otherwise it would be more costly in resources and time to reach those centres, which would then lead to a different weighting of results to take this additional imbalance into account. Bearing this in mind, the distribution shown in the column "Definitive Sample" in Table 2 was obtained, this distribution was used for the study.

The final procedure for sampling that was used as an alternative to simple random sampling based on census, requires two precautionary measures in the analysis phase. In the first place, as has already been mentioned, it is necessary to carry out an inverse probability weighting of the selection of each of the samples in each stratum; in the second place, a random effects model should be used, unless it is proven to be unnecessary, to adjust the sampling by schools, and within them by class and within them by pupils. Such precautions were taken into account as explained in the results section.

### Process of sample extracting

A letter was sent to the directors of all centres selected to ask them to transmit a request for permission to carry out the study to the School Board. Anonymity of the data of both individuals and centres was guaranteed.

Three weeks later, the centres were contacted again to set a date and to inform parents in case anyone refused to participate.

Data were collected by two scholarship holders who were specifically trained. On their arrival at each centre, using the lists that included birth dates and had been previously provided by the school, they determined the group of eligible pupils. Eligible pupils were those whose age was within the years or half years corresponding to their class ± 3 months. Of these pupils, four were selected in each class and one more as a possible substitute. If more were missing another child was chosen, but in no case more that two substitutes were required. Pupils with a non-Spanish parent were not eligible nor those with chronic illness or who were receiving a treatment known to affect their growth. For the purposes of the selection, a list of random numbers was used for each centre and for each day, prepared in relation to the different sizes of the eligible subpopulation.

In terms of data collection, incomplete weeks or weeks with exams were eliminated to avoid interrupting schoolwork or causing skewness due to absenteeism.

To obtain the second sample, for the group of 18- to 23-year olds, during the sampling in non-university education centres the sample was obtained in a similar manner to that of the sample of 3- to 18-year olds. In the case of university departments, after having received permission from the corresponding dean, we were able to use some time from the last main subject of the day. The information given to students was the same as in the other cases. In these situations, the volume of students who refused to participate was high, in many cases over 40%, having identified these data visually by subtracting those who were originally in class and those who were measured in the end. Data were collected by the same observers and using the same methodology and instruments as in the other samples. The subjects included were healthy, not suffering from chronic illness or undergoing continuous medical treatment that might affect their growth, they were born in Andalusia, were Caucasian and of Spanish origin.

In any case, this "extended" sample of 18- to 23-year olds cannot be considered representative of the Andalusian population and, in the best of cases, it is obvious when it does not differ significantly from the part of the sample correctly selected that overlaps with it and we can conjecture from this, if it proves to be the case, that differences are not great.

## Methods

### Personnel

Data collection was carried out by two scholarship holders who received special training. They were trained in techniques for weighing and measuring the sample being studied. Moreover, in all cases, measurements were taken by the same person.

Error in measurement was determined based on the measurements of 5 children repeated three times, as the standard error of the mean measurement of each child, giving a value of 0.2 cm.

Data were collected from February to December 2004 for the sample of up to the age of 18. For the broader sample, data were collected from January to May 2005, except February when there were no classes.

### Parameters studied and technical equipment used

A portable electronic Seca weighing machine, with an accuracy of 100 gr and automatic reset to 0, was used for weighing.

A portable Holtain stadiometer (height rod), with an accuracy of 0.1 cm was used for measuring height. Before each measuring session it was adjusted with an unbendable 65 cm rod.

The Body Mass Index was calculated using the following formula:

$$BMI=\frac{Weight(Kg)}{Height^{2}{\left(m\right)}^{2}}$$

### Measuring techniques

The pupils selected were asked to confirm that they gave their permission to be weighed and measured, and they were informed that if they preferred, measurement and weighing could be done without the presence of other pupils, that they did not need to undress, their data would not be read out and they would remain anonymous. Information was not provided to the next class unless all pupils for the previous class had been measured.

They were asked to take off their shoes and coats, if they were wearing one. A register of what each pupil was wearing was made, and this was grouped into three categories: light, medium and heavy. These categories were identified size by size in a department store and weighed so that the mean weight of the clothes for each age could be subtracted. A weight of between 300 gr and 900 gr was subtracted for each pupil. This methodological approach has been used in similar studies, in order to reduce the refusal rate. Particularly problematic is the group of older students, where a high refusal rate is expected. In this case the approach was used to reduce refusal due to the need for unclothing in the presence of peers.

With regards to height, using the portable Holtain stadiometer the children stood without shoes so that their heels, glutei and scapulae were in contact with the vertical plane and their heads were leaning against the so-called Frankfurt plane. With their ankles together, their inner malleola touching, and the soles of their feet firmly placed on the hard horizontal plane, the observer gently pushed their mastoid bones upwards. In this position, they were asked to breathe in deeply and the observer measured their height pushing down the mobile top to minimize to the maximum any error due to hair thickness.

A sheet was prepared to write down data on paper format, which was later recorded on electronic format, on a spreadsheet, within a maximum of 48–72 hours. Data were periodically revised to detect and correct any recording errors.

### Statistical methods

#### Statistical methods for adjusting Reference Curves

We used Cole's LMS method [21], which models the relationship between percentiles and age using a regression technique and assumes a normal distribution of the transformed variable.

This is a widely used method, especially in Europe, for the creation of reference tables depending on age; moreover, computer software in standard statistical packages is also available.

The method assumes that, in each age group, the anthropometric data can be adjusted to a normal distribution after having been adequately transformed, taking into account the degree of asymmetry (L), central tendency (M) and dispersion (S).

Using the original data, for each moment of time (t) the following quantities are obtained:

• L(t) value of the parameter *λ *of the transformation of Box-Cox to obtain the normality of the variable.

• M(t) median of the original data in the t instant.

• S(t), coefficient of variation of the original data in the t instant.

Obtained for the different t values, within the time frame considered, they are adjusted, using a penalized likelihood, a method that connects them with age.

Using the formula that appears below (and that is simply the result of undoing the change applied in order to normalize the variable) the *α *percentile is calculated for the t instant, which is given by this expression:

*C*_*α*_(*t*) = *M*(*t*)(1+*L*(*t*)*S*(*t*)*z*_*α*_)^1/*L*(*t*)^

where z_*α *_is the value of the function of an N (0.1) distribution that leaves to its left a *α *probability.

What Cole's method does is to model the skewness and the kurtosis of the variable (through the transformation that has to be made to convert the original variable into a Normal one), the central position of the variable (through the median) and the variance, and also the kurtosis in an indirect way (through the coefficient of variation of data in the t instant). Once these coefficients have been modelled in relation to time, thus obtaining the desired percentiles.

An assessment using the method of penalized likelihood requires specific statistical software. The STATA 8.1 and Splus S 6.0 packages were used.

#### Measuring the "goodness of fit"

The quality of the fit was assessed with tests proposed by Royston [22] that evaluate whether the model residuals follow a normal distribution based on their average, symmetry and kurtosis. Royston proposes a series of tests called Q-tests that characterize certain properties of the model residuals so that if the test yields a significant result it implies that the residuals do not adjust well to the normal random variable and the model does not adjust well either. They are based on the assumption that model residuals should be distributed according to an N (0.1) regardless time, if the model adjusts well.

The tests applied in the assessment of the goodness of fit were as follows:

• **Q**_1 _**test**: If the model adjusts well, the sum of the squares of the mean model residuals in each group, weighed by the size of the group, follows an *χ*^2 ^distribution with G-1 g.l.

• **Q**_2 _**test**: If the model adjusts well, the sum of a variances function in each model residuals group, follows an *χ*^2 ^distribution with G-1 g.l.

• **Q**_3 _**test**: If the model adjusts well, the sum of the squares of the experimental quantities from D'Agostino's Normality test for skewness, in each model residuals group, follows an *χ*^2 ^distribution with G g.l.

• **Q**_4 _**test**: If the model adjusts well, the sum of a function of the significance levels, P values, Shapiro-Wilks' Normality test, combined tests for skewness and Kurtosis, in each model residuals group follows an *χ*^2 ^distribution with 2 G g.l.

Each test responds to the imbalance in normality that the model can create. The first test concerns the differences between the residuals and the median of the distribution they should present, meaning, the **N **(0.1); the second test is related to a model residuals variance which is either too high or too low, that would suggest very or little sharp and therefore abnormal distributions;; the third test characterizes the skewness of the distributions of the model residuals that would indicate a way of non-normality of such distributions and therefore an imbalance; lastly, the fourth test refers at the same time to the skewness and kurtosis, which are two characteristics that mark non-normality. The latter could be significant for this study more easily, given that Cole's method does not directly model the kurtosis of the base distribution.

Royston suggests declaring the imbalance of the model when any of the tests proves to be significant at 5% error, a situation that rejects completely the adjusted model, given the great number of tests to be performed this is easy to happen simply thanks to the accumulation of errors in each one of them.

In our case, these tests were used to determine the number of edf necessary for each model's fit (Cole's LMS method), and we kept those models which achieved tests with more than 10% significance. When significant results were obtained in some tests, a study of the original observations was performed in case there were extreme data possibly contaminating the model to a great extent. The type of analysis applied is shown in the next section.

#### Method for detecting extreme and/or influential data

In the case in hand, the detection of extreme data should be carefully assessed. With the reference curves depending on time, our objective is to determine extreme values (high or low), thus eliminating extreme data from the sample could affect the curves in an obvious way, "pruning" the distribution of values that are valuable when it comes to determining percentiles. Nevertheless, in this kind of projects, it is not possible to leave out an analysis of extreme data given that this could entail an important risk of working with data which, almost certainly, do not belong to the target population.

There is a classic rule for detecting extreme data, which involves labelling a piece of data x as extreme in the following situations (Q(25) being the 25th percentile of the sample, Q(75) the 75th percentile and IQR the interquartile range):

x < Q(25) - 1,5 × IQR

x > Q(75) + 1,5 × IQR

We used, however, a modification to this rule, which makes it much more conservative:

x < Q(25) - 4 × IQR

x > Q(75) + 4 × IQR

With this modification we only detected data that were unusually extreme and in this way we do not risk "pruning" the distribution of data that could be influencing the tables.

Data are considered influential if, when eliminated from the sample, they generate a significant change in the model that is being adjusted, giving rise to a profoundly different one. Traditionally, influential data have been identified with extreme or very extreme data, although this is not always the case. A conservative, but reasonable, option would be to look for influential data only among the very extreme data in such a way that will not prejudice areas of distribution that are closest to the centre.

Assuming the previous considerations, the process for the detection and, when necessary, the elimination of data was as follows:

1) Extreme values were labelled for each age, using the rule explained above.

2) Taking labelled data as extreme into consideration, the model was adjusted and the tests were evaluated to determine whether they were significant or not. If this was not the case, it was acknowledged that the most extreme data, up to that moment, were not influential and the process was stopped, conserving all sample data. When the test was significant, the most extreme data were eliminated and the process was repeated until the adjustment quality control tests were not proven to be significant. The iterant process was always carried out on the most extreme data used.

We insist that this is a very conservative process, because this is required by the type of study we are carrying out and, as the results will show, it has led us to reject a very small number of observations.

### Analysis of the model's sensibility in terms of the lack of data for absent subjects

The elaboration of tables for height and weight assumes that the individuals included in the study do not suffer from chronic illness that could significantly affect their growth and weight gain. A screening, was performed to avoid including these boys and girls, although probably it was not very rigorous. This is taken into account in the final assessment and it is evaluated, as appropriate.

Those pupils who were absent on the day data were collected were substituted by others. As the number of pupils taken by class (between 4 and 6, in the most extreme case) was small, the volume of data substituted was not very large so the assessment of such values is not very efficient and it is not considered very relevant.

On the other hand, if absence from class was due to illness, this could be a factor generating a strong skewness on the results. A 2003 study provided by the provincial Education Delegations placed the mean daily rate of absenteeism in state schools in Andalusia at 12.8%. It could be argued that a big part of these absences were due to illness and that among the ill children it is more likely that there were some with chronic illness that might affect their growth. In this case, the sample would give a skewness in the tables towards values that are too "high".

An analysis was carried out of the sensibility of the final models using the least favourable scenario. Based on a the daily absenteeism level of 12.8%, it was assumed that 10% were, in relation to the variable of interest, below the median. Calculations were made based on this theoretical situation and the final results were compared with those obtained in reality. In this scenario, if curves did not change sufficiently, this meant that they were not affected by the skewness generated by non-measurements due to illness.

All these considerations are valid for the sample of 3- to 18-year olds. The other sample is subject to higher rates of absence of measurements and diverse possible skewness. Such skewness could strongly affect two characteristics of the distribution of variables: the width of values obtained and a skewness towards higher values in some variables and lower values in others. In the first case, if part of the extreme values had been eliminated we would have obtained tables that would have been too "narrow", which could be compared to the tables that resemble those of our population. In the second case, we would have skewness that would balance out but would be difficult to demonstrate or measure. For this reason, the same criterion of 10% illness, which is below the median, was applied to this sample. In the case of variables where the skewness acted inversely (weight and therefore BMI), such skewness has not been considered so the estimated figures would be, in that case, underestimated.

## Results

The total number of cases collected was 9,065; 50.07% were female (4,539 cases) and 49.93% were male (4,526 cases). Considering age, the group of up to 18 years of age made up 79.39% of the cases (7,197).

Of the 4,539 females included, 79.14% (3,592 cases) belonged to the group of up to 18-year olds, while of the 4,526 males, 79.65% (3,605 cases) were in the same age group.

The size of the sample of 3- to 18-year olds is larger than the one initially calculated, which could create a beneficial effect on the efficiency of the estimators. This increase in the sample is due to three reasons. In the first place, 5 male and 5 female pupils were measured in a significant number of classes instead of the 4 originally planned. Secondly, the number of units was higher than the mean estimated by the Education Board; and lastly, the total number of pupils used to make the preliminary estimations was from the previous year, while in several schools the number of children was higher than expected.

In relation to the sample of 3 to 18 year olds, none of the schools refused to participate and neither did any of the parents of the pupils. In this group, 3 women and 2 men, that is 0.07%, refused to participate. In 18 cases, children had some kind of prosthesis (plaster cast, corset) that did not affect there height; their weight was taken without any type of correction. Only 3 subjects from the sample (0.04%) were receiving endocrinological treatment and they were not eliminated from the sample.

In the sample of 3- to 18-year olds, 327 pupils were substituted because they were not present when the sample was taken from their class; they represent 4.5% of this part of the sample. This figure is so low that it does not seem useful for estimating absence, and this could be due to several reasons. On the one hand, the weeks chosen did not contain events that might reduce pupils' attendance (local holidays, bank holidays, etc...), while Mondays, which is the day with the highest rate of absenteeism, was also excluded because it was the day the personnel chose to travel to each centre. On the other hand, since teachers were given a list of the selected pupils beforehand, it is possible that the most 'work-inclined' ones may have caused an indirect skewness by not "remembering" that they had substituted pupils who were not present. This last skewness was not detected until the study was well underway so it could not be corrected and, although it is probably low, it forced us to carry out an analysis of the robustness of the results against this skewness, as has already been mentioned.

The finally selected sample of the population aged between 18 and 23 (1,868 cases) is slightly smaller than that initially planned. Nevertheless, the difference of 132 people is small and should not have generated skewness on the results beyond the one that had already affected this part of the sample and have already been mentioned in the methodology section.

In the sample of over 18-year olds, the sampling technique does not allow such an accurate analysis of the general results. Based on direct estimates, in some cases it is known that the non-response rate might have reached up to 40% which makes us suspect of some significant skewness; in any case, as was stated in the statistical method section, sensibility studies will be performed applying the tables to these facts.

The distribution of the sample of men and women by ages, grouped by years and half years is shown in Table 3.

**Table 3:** Distribution of the men and women sample by age.

	**WOMEN**			**MEN**		
**Age (years)**	**Subjects no.**	**Percentage**	**Accumulated percentage**	**Subjects no.**	**Percentage**	**Accumulated percentage**

3	90	1.98	1.98	97	2.14	2.14
3.5	134	2.95	4.94	132	2.92	5.06
4	126	2.78	7.71	145	3.20	8.26
4.5	112	2.47	10.18	134	2.96	11.22
5	114	2.51	12.69	116	2.56	13.78
5.5	125	2.75	15.44	116	2.56	16.34
6	104	2.29	17.74	108	2.39	18.73
6.5	122	2.69	20.42	131	2.89	21.62
7	116	2.56	22.98	112	2.47	24.09
7.5	144	3.17	26.15	132	2.92	27.01
8	132	2.91	29.06	108	2.39	29.40
8.5	110	2.42	31.48	132	2.92	32.32
9	126	2.78	34.26	129	2.85	35.17
9.5	111	2.45	36.7	124	2.74	37.91
10	121	2.67	39.37	135	2.98	40.89
10.5	131	2.89	42.26	130	2.87	43.76
11	141	3.11	45.36	124	2.74	46.50
11.5	133	2.93	48.29	135	2.98	49.48
12	122	2.69	50.98	109	2.41	51.89
12.5	138	3.04	54.02	111	2.45	54.34
13	117	2.58	56.6	135	2.98	57.32
13.5	123	2.71	59.31	116	2.56	59.88
14	97	2.14	61.45	104	2.30	62.18
14.5	95	2.09	63.54	100	2.21	64.39
15	112	2.47	66.01	99	2.19	66.58
15.5	90	1.98	67.99	89	1.97	68.55
16	110	2.42	70.41	96	2.12	70.67
16.5	119	2.62	73.03	101	2.23	72.90
17	81	1.78	74.82	85	1.88	74.78
17.5	83	1.83	76.65	96	2.12	76.90
18	113	2.49	79.14	124	2.74	79.64
18.5	70	1.54	80.68	90	1.99	81.63
19	72	1.59	82.26	58	1.28	82.91
19.5	104	2.29	84.56	111	2.45	85.36
20	107	2.36	86.91	88	1.94	87.30
20.5	97	2.14	89.05	92	2.03	89.33
21	107	2.36	91.41	95	2.10	91.43
21.5	96	2.12	93.52	86	1.90	93.33
22	110	2.42	95.95	116	2.56	95.89
22.5	110	2.42	98.37	105	2.33	98.22
23	74	1.63	100.0	80	1.77	100.0

TOTAL	4,539	100		4,526	100	

### Height

#### Sample data

A summary of the height data in the samples of men and women is shown in Table 4.

**Table 4:** Stature values (mean and standard deviation) in the samples of men and women.

	**WOMEN**			**MEN**		
**Age (years)**	**Subjects no.**	**Mean stature (cm)**	**Standard deviation**	**Subjects no.**	**Mean stature (cm)**	**Standard deviation**

3	90	96.5	4.07	97	97.7	3.77
3.5	134	98.3	3.98	132	99.9	3.79
4	126	102.0	4.18	145	103.1	3.99
4.5	112	106.2	3.83	134	106.5	4.41
5	114	109.3	4.93	116	109.9	4.59
5.5	125	112.4	4.93	116	113.1	4.48
6	104	116.3	5.45	108	117.0	5.27
6.5	122	119.0	6.57	131	121.2	5.08
7	116	121.4	4.52	112	122.3	5.64
7.5	144	125.2	5.09	132	125.7	5.90
8	132	126.5	6.18	108	128.8	5.51
8.5	110	131.3	5.77	132	132.5	6.03
9	126	132.8	6.57	128	134.1	5.35
9.5	111	135.6	6.53	124	136.7	5.86
10	121	139.3	6.62	135	140.0	6.04
10.5	131	142.5	7.25	130	141.7	6.82
11	141	146.2	7.21	124	144.4	6.80
11.5	133	148.9	7.07	135	146.9	7.64
12	122	151.5	6.56	109	151.4	7.74
12.5	138	154.8	6.84	111	154.9	7.95
13	117	156.3	5.87	135	156.5	8.43
13.5	123	158.0	6.25	116	159.9	8.66
14	97	158.5	5.84	104	163.2	8.67
14.5	95	158.6	5.68	100	167.0	6.46
15	112	161.1	6.06	99	170.8	7.10
15.5	90	160.7	5.78	89	171.7	6.77
16	110	161.3	5.62	96	172.1	6.30
16.5	119	161.1	6.51	101	173.1	6.40
17	81	160.7	5.37	85	175.0	6.37
17.5	83	163.0	5.72	96	174.5	6.67
18	113	162.6	5.51	124	174.9	6.37
18.5	70	163.7	6.45	89	173.9	6.29
19	72	161.7	6.19	58	175.8	6.89
19.5	104	162.6	5.56	111	175.9	6.85
20	107	162.2	5.13	88	175.4	6.98
20.5	97	162.7	7.03	92	176.3	6.75
21	107	163.1	5.79	95	176.2	7.35
21.5	96	164.2	6.16	86	176.7	6.59
22	110	162.9	5.87	116	176.5	6.54
22.5	110	163.7	5.79	105	176.0	6.91
23	74	163.4	6.55	80	176.8	6.91

It can be appreciated that in the sample of women the approximate height value becomes stable as of the age of 18, while in men, it stabilizes beyond the age of 20. For this reason, comparisons of mean heights were performed for age groups calculated every half year beyond the above-mentioned values. The results obtained in both cases (women F_exp _= 1.36 (10; 1049) g.l. p = 0.1938 and men F_exp _= 0.44 (6; 655) g.l. p = 0.8496) confirm the impression that the average height does not vary significantly beyond these ages. As a result the values beyond the age of 20 were grouped together, in the sample of women and men, to refer to the Andalusian adult height in each gender.

#### Extreme values

Following the method already explained in the methodology section, the values considered extreme were categorized. In the women's group, 1 three cases were corrected because they contained automation errors that had not been detected during the first phase of corrections. Another two were kept because they were not proven significant during the quality adjustment tests.

On the contrary, the two cases detected as extreme in the men's group were eliminated because, when they were included, they were proven significant during the quality adjustment tests.

#### Model adjustment

**Figure 1:**
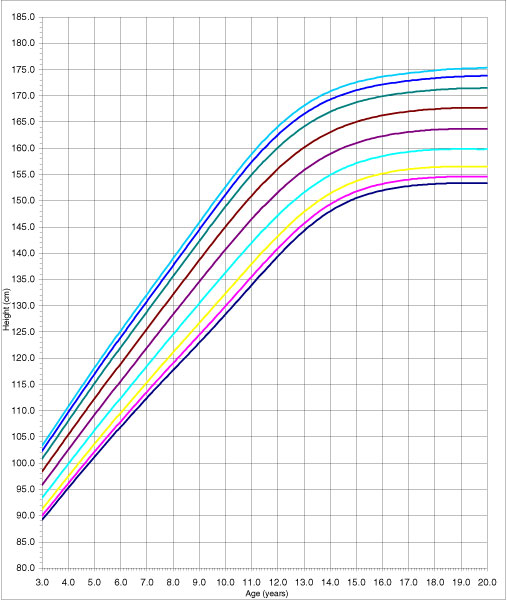
The 3, 5, 10, 25, 50, 75, 90, 95 and 97 percentile curves for women's stature.

**Figure 2:**
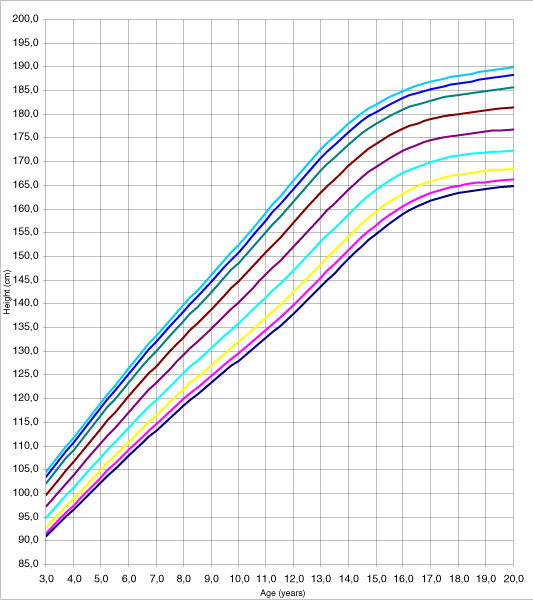
The 3, 5, 10, 25, 50, 75, 90, 95 and 97 percentile curves for men's stature.

The adjustment of the model can be considered adequate since none of the tests used (Q1, Q2, Q3, and Q4) yielded significant results. Adjustment was analysed using a random effects model (the classroom was the grouping unit for the 4–6 pupils chosen in it); following the performance of the likelihood ratio test to compare with the fixed effects model the values obtained were *χ*^2^_exp _= 1.63 (1 g.l.) p = 0.2014 for women and *χ*^2^_exp _= 1.08 (1 g.l.) p = 0.3102 for men. The use of the fixed effects model adjustment, produced the height table for men and women, in decimals of years from the age of three to 19 concentrating the values beyond this in 20 (Additional data file 1: Table A and B). Figures 1 and 2 present percentiles 3, 5, 10, 25, 50, 75, 90, 95 and 97 for height, of both men and women.

#### Sensitivity analysis

As explained in the methods section, a sensitivity analysis was performed on the final model using the most unfavourable scenario. To this end, it was assumed that 10% of the individuals measured could not be measured, and that all of them were below the median. Then, when we compared this hypothetical situation to the real model, we found that for estimating the median, in the worst case (around the age of ten) the difference in the tables would be 8 mm in women and 9 mm in men, an amount that seems small. For the most important situation, from a clinical point of view, which would be that of the percentile 3, the maximum difference was half a centimetre, assuming that 10% of the children were not measured and that all of them were below median measured.

### Weight

#### Sample data

Table 5 summarizes data for weight in men and women. In women, weight stabilizes by the age of 18. To confirm this fact, we compared the mean weight in age groups of half years beyond this age (F_exp _= 1.36 (10;, 1049) g.l. p = 0.1952). Performing the same comparisons in the men's sample, the mean weight had not been stabilized beyond the age of 18 (F_exp _= 2.15 (10; 1054) g.l. p = 0.0188), but it had done so in age groups above 20 (F_exp _= 1.58 (6; 655) g.l. p = 0.1514). For this purpose, in the sample of men and women, the values beyond the age of 20 were grouped together so as, to refer to the weight of Andalusian adults of each gender.

**Table 5:** Weight values (mean and standard deviation) in the samples of men and women.

	**WOMEN**			**MEN**		
**Age (years)**	**Subjects no.**	**Mean weight (kg)**	**Standard deviation**	**Subjects no.**	**Mean weight (kg)**	**Standard deviation**

3	90	14.9	1.70	97	15.7	3.78
3.5	134	15.7	2.65	132	16.1	2.36
4	126	17.0	2.85	145	17.3	2.59
4.5	112	18.1	2.61	134	18.3	3.11
5	114	19.1	3.38	116	19.6	3.26
5.5	125	20.5	4.23	116	21.2	4.03
6	104	22.7	4.41	108	22.8	4.04
6.5	122	24.3	5.17	131	25.1	4.88
7	116	25.5	5.67	112	25.7	6.28
7.5	144	27.3	6.19	132	27.6	6.53
8	132	28.2	6.86	108	28.8	5.48
8.5	110	32.0	7.74	132	33.0	9.16
9	126	33.3	8.17	129	33.8	8.54
9.5	111	35.7	8.85	124	35.6	8.54
10	121	37.0	9.92	135	37.9	9.60
10.5	131	39.8	9.87	130	39.8	11.79
11	141	43.9	11.11	124	43.4	12.44
11.5	133	45.6	12.28	135	43.3	11.63
12	122	48.1	11.23	109	47.4	11.78
12.5	138	51.2	14.07	111	51.3	14.31
13	117	53.2	11.98	135	51.1	12.13
13.5	123	54.1	12.10	116	54.2	12.50
14	97	55.0	12.14	104	58.0	13.64
14.5	95	56.4	11.27	100	60.1	12.82
15	112	57.2	13.24	99	67.1	13.61
15.5	90	58.1	9.87	89	66.2	13.16
16	110	56.8	9.89	96	67.5	13.86
16.5	119	57.8	10.44	101	67.8	13.37
17	81	56.5	9.45	85	70.1	12.95
17.5	83	58.8	9.48	96	70.5	12.71
18	113	57.6	8.94	124	70.5	14.20
18.5	70	57.3	9.74	90	70.9	14.51
19	72	57.4	7.99	58	74.3	14.25
19.5	104	59.9	10.41	111	73.2	11.10
20	107	57.0	8.84	88	73.1	10.46
20.5	97	58.5	9.08	92	74.9	11.76
21	107	57.2	7.93	95	71.9	10.61
21.5	96	58.6	7.78	86	74.6	10.93
22	110	57.0	7.48	116	73.1	10.24
22.5	110	59.7	11.76	105	74.7	11.50
23	74	57.9	8.89	80	76.3	12.62

#### Extreme values

All values, from both samples that were characterized as extreme (5 in women and 6 in men) were eliminated since the goodness of fit test was proven significant in case they were included, and they had a considerable effect on the kurtosis of the distribution.

#### Model adjustment

**Figure 3:**
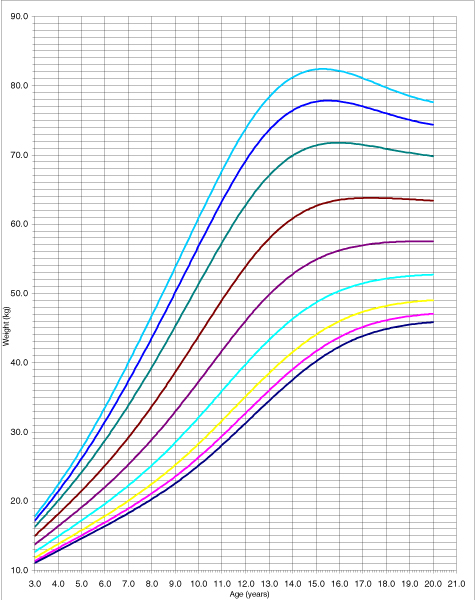
The 3, 5, 10, 25, 50, 75, 90, 95 and 97 percentile curves for women's weight.

**Figure 4:**
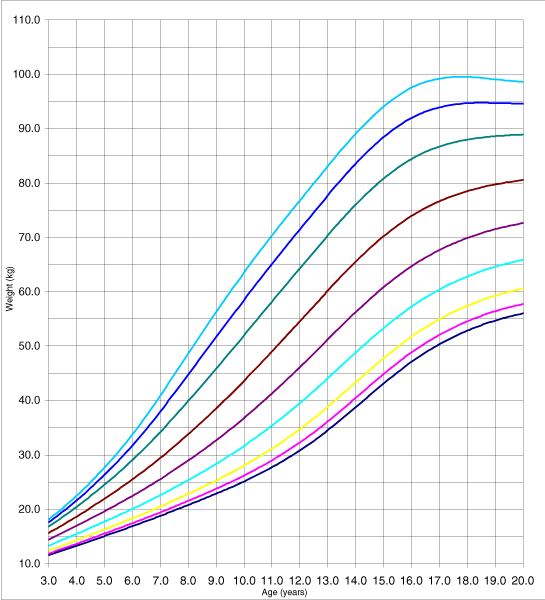
The 3, 5, 10, 25, 50, 75, 90, 95 and 97 percentile curves for men's weight.

The adjustment is adequate because none of the tests assessing its goodness of fit (Q1, Q2, Q3, and Q4) yielded a significant result. The adjustment was analysed using a random effects model (the classroom was the grouping unit for the 4–6 pupils selected in it); following the performance of the likelihood ratio test to compare with the fixed effects model, the values obtained were *χ*^2^_exp _= 1.08 (1 g.l.) p = 0.2987 for women and *χ*^2^_exp _= 1.49 (1 g.l.) p = 0.2222 for men. The use of the fixed effects model adjustment, produced the height table for men and women, in decimals of years from the age of three to 19 concentrating the values beyond this in 20 (Additional data file 1: Tables C and D). Figures 3 and 4 show percentiles 3, 5, 10, 25, 50, 75, 90, 95 and 97 for weight, in both women and men.

#### Sensitivity analysis

In the same way as with height, a sensitivity analysis of the final models was performed, using the most unfavourable scenario. To this end, it was assumed that 10% of the individuals weighed could not be weighed, and that all of them were below the, median. Then, when we compared this hypothetical situation with the real model, we found that for the estimation both of the median and of the percentile 3, differences in women were between 0.1 and 0.8 kg (around the age of 14). In the case of men, differences in the estimation of the median ranged between 0.1 and 1 kg, while in the case of the percentile 3 which has the greatest clinical significance, it ranged between 0.1 and 0.3 kg.

### Body Mass Index (BMI)

#### Sample data

The BMI data in the men and women's samples are shown in Table 6. For this reason, comparisons were performed of the BMI mean values for ages over 18 grouped by half years, thus confirming that it does not change significantly as of that age either in men or women (women: F_exp _= 1.25 (10; 1049) g.l. p = 0.2557 and men: F_exp _= 1.30 (10; 1034) g.l. p00.2264). In the case of BMI, the values for the ages over 20s were also grouped together in the sample of men and women, so as to refer to the BMI of adult Andalusians in each gender.

**Table 6:** BMI values (mean and standard deviation) in the samples of men and women.

	**WOMEN**			**MEN**		
**Age (years)**	**Subjects no.**	**Mean BMI (kg/m^2^)**	**Standard deviation**	**Subjects no.**	**Mean BMI (kg/m^2^)**	**Standard deviation**

3	90	16.0	1.28	97	16.4	3.00
3.5	134	16.1	1.92	132	16.1	1.69
4	126	16.2	2.01	145	16.2	1.90
4.5	112	16.0	1.84	134	16.1	2.07
5	114	15.9	2.06	116	16.2	1.83
5.5	125	16.1	2.28	116	16.5	2.51
6	104	16.7	2.61	108	16.6	2.50
6.5	122	17.1	2.88	131	17.0	2.67
7	116	17.2	3.13	112	17.0	3.12
7.5	144	17.3	3.13	132	17.3	3.16
8	132	17.5	3.48	108	17.3	2.77
8.5	110	18.4	3.67	132	18.6	3.95
9	126	18.7	3.49	129	18.6	3.85
9.5	111	19.2	3.53	124	18.9	3.60
10	121	18.9	4.13	135	19.1	3.80
10.5	131	19.5	4.03	130	19.6	4.69
11	141	20.5	4.56	124	20.6	4.62
11.5	133	20.4	4.52	135	19.8	4.04
12	122	20.8	3.90	109	20.5	4.11
12.5	138	21.2	5.29	111	21.2	4.84
13	117	21.7	4.34	135	20.7	4.10
13.5	123	21.6	4.37	116	21.1	3.86
14	97	21.9	4.30	104	21.6	4.06
14.5	95	22.4	4.15	100	21.5	4.03
15	112	21.9	4.45	99	23.0	4.27
15.5	90	22.5	3.45	89	22.4	4.06
16	110	21.8	3.41	96	22.7	4.05
16.5	119	22.2	3.48	101	22.6	4.04
17	81	21.8	3.30	85	22.8	3.72
17.5	83	22.1	3.46	96	23.1	3.74
18	113	21.8	3.17	124	23.0	4.37
18.5	70	21.4	3.33	90	23.5	4.24
19	72	22.0	3.13	58	24.1	4.69
19.5	104	22.1	3.62	111	23.6	3.46
20	107	21.1	3.21	88	23.7	3.38
20.5	97	22.0	3.04	92	24.0	3.38
21	107	21.4	2.71	95	23.5	2.99
21.5	96	21.5	2.84	86	24.1	3.23
22	110	21.2	2.81	116	23.5	2.60
22.5	110	22.0	4.04	105	24.2	3.39
23	74	21.4	2.76	80	24.4	3.19

#### Extreme values

Both in the case of men and women, all values characterised as extreme were eliminated since the tests showed that they significantly affected the quality of adjustment. 13 cases were eliminated in the women's sample (0.29% of the sample) and 8 in the men's group (0.18% of the sample).

#### Model adjustment

**Figure 5:**
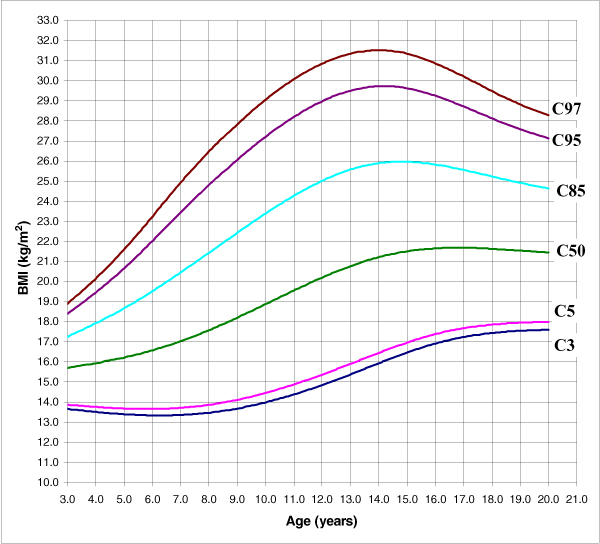
The 3, 5, 50, 85, 95 and 97 percentile curves for women's BMI.

**Figure 6:**
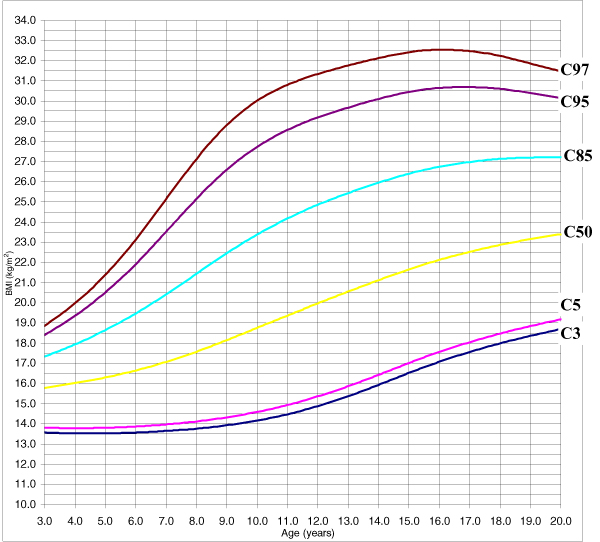
The 3, 5, 50, 85, 95 and 97 percentile curves for men's BMI.

The adjustment of the model is adequate given that none of the tests evaluating the goodness of fit (Q1, Q2, Q3, and Q4) yielded a significant result. The adjustment was analysed using a random effects model (the classroom was the grouping unit for the 4–6 pupils chosen in it); following the performance of the likelihood ratio test to compare with the fixed effects model the values obtained were *χ*^2^_exp _= 2.37 (1 g.l.) p = 0.1237 for women and *χ*^2^_exp _= 2.21 (1 g.l.) p = 0.1371 for men. The use of the adjustment of the fixed effects model produced the BMI table for women and men, grouped in ages of half years from the age of three to 19, with all ages over 20 concentrated at 20 (Additional data file 1: Table E). Figures 5 and 6 show percentiles 3, 5, 50, 85, 95 and 97 for BMI, in both women and men. These are the percentiles considered both in the BMI table and graphs since from a clinical point of view the most useful values are those corresponding to percentiles 3, 50, 85 and 95.

With regards to the BMI, the evolution of the percentiles below the median is very different from the evolution of the percentiles that are above such median for both genders, especially that of the most extreme percentiles. In the women's model, percentiles 85, 95 and 97 increase significantly until the age of 14, when they start to decrease. So Andalusian girls from the age of 8 or 9 have a higher probability of being overweight or obese and this probability reaches its maximum at the age of 14, when it starts to diminish. In the case of men, percentiles 95 and 97 show a similar evolution, although they reach their peak around the age of 16.

#### Sensitivity analysis

In the sensitivity analysis of the final BMI model, it was also assumed that 10% of the individuals measured were not actually measured, and that the BMI values of all of them were below the median. This way, when we compared this hypothetical situation with the real model, we found that for the estimation of the median and of the percentile 85, both in the sample of men and women, differences ranged between 0.1 and 0.3. These differences are noticeably lower in the extreme percentiles, both above and below the median.

## Discussion

This work presents the results of the first population study carried out in Andalusia to discover the weight, height and BMI values of individuals between the ages of 3 and 23.

We have carried out a cross sectional study because this allows to obtain applicable results in a short period of time; in the second place, the cost was significantly lower than that of a longitudinal study. Moreover, if the data obtained were not different to that of the longitudinal study performed in Aragon, which proved to be the case, the disadvantages of a transversal study could be compensated for by using the data on growth rate and puberty provided in that study.

Our study is the broadest of those developed in the Andalusian population and we have worked with a random sample, selected via a multistage sampling, representative of the young population of Andalusia with a total sample of more than 9,000 subjects.

We should emphasize that, despite the sample size, all measurements were taken, using precise instruments, by the same, properly trained examiner. This contributes to increasing the internal validity of our study. Moreover, with the aim of avoiding repetitions and other errors, the data were registered in duplicate on a database which was revised centrally at intervals of less than a week.

It is worth mentioning the scarcity of non-participation. In the first place, the fact that no school (public or private) refused to take part is a relevant fact and denotes the level of collaboration of schools in terms of health issues. In the second place, the fact that no parents refused to allow their children to participate could be because they do not perceive the collection of data as offensive or harmful for their children; or this could mean that schools failed to inform them about the process. Nevertheless, no centre has contacted us since to communicate any complaints from parents. We think that the children's almost anecdotal refusal to be measured is the result of keeping them continuously informed about the process and the care with which data were collected. Privacy and anonymity during measurements were especially important, because although it was more time-consuming, it increased pupils' confidence. A small group of pupils (0.6%), most of them female, had some objections about their being measured, but in the end they agreed to participate. In the majority of these cases the origin of the problem was that they were overweight. This suggests that we avoided a skewness that could have produced if we had not been able to collect such data. Moreover, we are convinced that if these subjects had refused, they would have also encouraged others to do the same, in case of a lack of ideal conditions of privacy and anonymity. We believe that an immediate consequence of such a high level of collaboration on the part of schools, parents and pupils shows that if projects are well designed with adequate precaution, education centres can be places for obtaining samples of sufficient quality to substitute simple random samples based on census, thus saving resources.

It was necessary to substitute 327 of the selected pupils because they were absent from school when data were collected. This figure represents 3.7% of the pupils in the final sample, which is a low number, so it is not useful as an estimation of absenteeism on sampling days. There are several facts that contribute to such a low level of absence. As mentioned earlier, data were collected in weeks without foreseeable events (there were no bank holidays, local holidays or general exams, etc) and, in general, data were not collected on Mondays (the day of the week with the highest rate of school absenteeism) because this was the day used for travelling to the centres. Another reason is actual dynamics of the data collection process whereby a list of the pupils selected for the sample was given to the teacher in advance so they could inform them. This way, we discovered that the most work-inclined teachers could be causing an indirect skewness on the sample by not "remembering" that they had substituted pupils who were not present. This last skewness was not detected until the study was well underway so it could not be corrected and, although it could not have been great, it led us to carry out an analysis of the "robustness" of the results to this skewness, as mentioned in the statistical method section.

In summary, with regards to the sample of 3 to 18 year olds, we had a larger sample than anticipated in relation to the young Andalusian population. The non-participation rate among the subjects selected was very low as was the level of non-participation due to absence, although it was not possible to calculate it with precision, we have been able to check that the results are sufficiently robust in the face of this potential skewness.

In the sample of the young population between the ages of 18 and 23, despite the lower quality of the statistical parameters estimation in the population of origin, it was assumed that the great variance in the parameters measured in this group of population would ensure a representation of extreme values, even though the sample was not random. Moreover, this type of sampling is not unusual in studies similar to this one; Van Buuren [23] explains a similar way of broadening the sample size beyond the age of 17 in these cases, although with an extremely high rate of non-participation.

In the sensitivity analysis of the final model, we were able to confirm that the tables obtained are fairly robust against significant fluctuations and skewness. So much so that even assuming that 10% of the subjects had not been measured and that all of them were below the median, the percentile 3 would not have differed by more than half a centimetre. Differences of similar importance could have also occurred in the rest of the parameters analysed, as was shown in the results section.

When we compare our results with those of other authors of contemporary Spanish projects, it is observed that in terms of height there is very little difference in the percentile 50. Mean height in these studies (Bilbao, Enkid, Zaragoza and Barcelona) for men ranges from 176.3 to 177.7 cm, with our study being 176.7 cm. Women's height ranges from 162.1 to 165 cm while that of our study was 163.7 cm. Nevertheless, during growth in intermediary age groups there are important differences which are probably related to growth during puberty ('Proyecto Crece' ['Grow Project'], unpublished data). In terms of weight and BMI, the distribution is distanced from normality with a curve deviation towards the high percentiles above the median. This situation is also reflected in the Spanish studies given the increasing prevalence of weight problems in our population. These data, although reflecting the situation of the population, cannot be considered as a reference for our children because they represent an overweight population. To this end, we should use as a reference the patterns that take into account overweight or obesity cut-offs in adults (25 kg/m^2 ^and 30 kg/m^2^). Cole's study [24] was performed using samples from six countries and almost 100,000 subjects of each sex aged between 0 and 25. The objective was to establish the cut-offs for each age, based on the percentiles where the values of 25 kg/m^2 ^(for overweight) and 30 kg/m^2 ^(for obesity) were situated at the age of 18. Other studies have compared the use of cut-offs with percentiles 85 and 97 for each ages group [25], without revealing any difference in prevalence for overweight, unlike the case of obesity calculation. When we used the cut-off values established by Cole as a diagnostic criterion, the women in our study increased the ponderal excess (overweight plus obesity) from the age of 4 (25.1%) to 9 (40%) falling to 15.7% at the age of 18. The maximum overweight was 27.4% for 10,5-year olds, while that of obesity was 14.5% for 7-year olds. At 18, the corresponding indexes were 13.4% and 2.3% respectively.

Comparing to Cole's cut off values as well, the males in our study increase their ponderal excess (overweight plus obesity) from the age of 4 (19.3%) to 9 (38.8%) falling to 28.4% at the age of 18. The maximum level of overweight was 26.8% at the age of 12, while that of obesity 14% at the age of 8. At 18, the corresponding indexes were 22.6% and 5.9% respectively.

Our data coincide with a study on the prevalence of obesity in Spain carried out by the Spanish Society for the Study of Obesity (Sociedad Española para el Estudio de la Obesidad/SEEDO), which analysed several studies all over Spain, and quantified the ponderal excess in men at 29.5% and women at 19.1% [26]. The difference in sexes appears in all the studies, with women showing lower ponderal excess than men, probably due to their greater concern for their physical appearance at these ages.

The differences found as compared to other Spanish studies are mostly methodological. This is the broadest and most extended transversal study, based completely on a random sampling process, except for the adjustments applied to very small populations. Therefore, in our opinion, it is the most representative one in terms of the population studied (about 8 million inhabitants).

Limitations mostly affect the sample of 18 to 23 year olds, which only took place in two cities and only included university population. Taking into account that this method is used by other authors and this is the only really accessible population, we should also consider that there are unpublished data from a study in Cordoba, where the average height of male university students exceeded the non-university population by 1.8 cm and that of women by 0.9 cm; both in a significant way (unpublished data).

After this study, we are still lacking reference standards for the Andalusian infant population under 3. This stage of life is particularly important given the vulnerability of the health of infants and young children. Therefore, an adequate assessment of growth in height and weight are indicators of their health status, and even the socio-economic development of the communities they live in.

Similarities among various Spanish studies carried out in this century using similar methodology suggest that we should probably integrate them all into a single national reference pattern and combine them so as to create Spanish reference tables, like the UK90 tables created in the United Kingdom [4].

## Funding

The project was financed by a project of the Andalusian Health Service (Servicio Andaluz de Salud) (137/04) and a research grant from Imabis Foundation to cover field researchers' fees.

## Competing interests

The authors declare that they have no competing interests.

## Authors' contributions

JPLS y JMFG have contributed similarly to the study concept and design, acquisition of data, analysis and interpretation of data, and writing of the manuscript. JAMM has contributed to the analysis and interpretation of data, and the writing of the manuscript. JDLC has designed the sampling maethod and made the satistical analysis of data. CRC and AJO similarly contributed to the discussion of the manuscript.

## Supplementary Material

Additional file 1 **Tables of stature, weight and BMI for men and women**. Table A. Women's stature; Table B. Men's stature; Table C. Women's weight; Table D. Men's weight; Table E. Women's and men's BMI. Click here for file
