# Supplementary material for: Cross-sectional study of height and weight in the population of Andalusia from age 3 to adulthood
Source: BMC Endocr Disord. 2008 Jul 18;8(Suppl 1):S1. doi: 10.1186/1472-6823-8-S1-S1 (PMC2493384; doi:10.1186/1472-6823-8-S1-S1)
Supplement: Additional file 1 — Tables of stature, weight and BMI for men and women. Table A. Women's stature; Table B. Men's stature; Table C. Women's weight; Table D. Men's weight; Table E. Women's and men's BMI. [file 1472-6823-8-S1-S1-S1.doc]

**TABLE A. Women's stature**

| **Stature percentiles for women** | | | | | | | | | |
| --- | --- | --- | --- | --- | --- | --- | --- | --- | --- |
| **Age** | **P3** | **P5** | **P10** | **P25** | **P50** | **75** | **P90** | **P95** | **P97** |
| **3.0** | 89.2 | 90.0 | 91.2 | 93.4 | 95.8 | 98.4 | 100.8 | 102.3 | 103.3 |
| **3.1** | 89.8 | 90.6 | 91.8 | 94.0 | 96.5 | 99.1 | 101.5 | 103.0 | 104.0 |
| **3.2** | 90.4 | 91.2 | 92.5 | 94.6 | 97.2 | 99.8 | 102.2 | 103.8 | 104.8 |
| **3.3** | 91.0 | 91.8 | 93.1 | 95.3 | 97.8 | 100.5 | 103.0 | 104.5 | 105.5 |
| **3.4** | 91.6 | 92.4 | 93.7 | 95.9 | 98.5 | 101.2 | 103.7 | 105.2 | 106.3 |
| **3.5** | 92.2 | 93.0 | 94.3 | 96.6 | 99.2 | 101.9 | 104.4 | 106.0 | 107.0 |
| **3.6** | 92.8 | 93.6 | 95.0 | 97.2 | 99.8 | 102.6 | 105.1 | 106.7 | 107.7 |
| **3.7** | 93.4 | 94.3 | 95.6 | 97.9 | 100.5 | 103.3 | 105.9 | 107.4 | 108.5 |
| **3.8** | 94.0 | 94.9 | 96.2 | 98.5 | 101.2 | 104.0 | 106.6 | 108.2 | 109.2 |
| **3.9** | 94.6 | 95.5 | 96.8 | 99.2 | 101.9 | 104.7 | 107.3 | 108.9 | 110.0 |
| **4.0** | 95.2 | 96.1 | 97.5 | 99.8 | 102.5 | 105.4 | 108.0 | 109.7 | 110.7 |
| **4.1** | 95.8 | 96.7 | 98.1 | 100.5 | 103.2 | 106.1 | 108.7 | 110.4 | 111.5 |
| **4.2** | 96.4 | 97.3 | 98.7 | 101.1 | 103.9 | 106.8 | 109.5 | 111.1 | 112.2 |
| **4.3** | 97.0 | 97.9 | 99.3 | 101.7 | 104.5 | 107.4 | 110.2 | 111.8 | 113.0 |
| **4.4** | 97.6 | 98.5 | 99.9 | 102.4 | 105.2 | 108.1 | 110.9 | 112.6 | 113.7 |
| **4.5** | 98.2 | 99.1 | 100.6 | 103.0 | 105.9 | 108.8 | 111.6 | 113.3 | 114.4 |
| **4.6** | 98.8 | 99.7 | 101.2 | 103.7 | 106.5 | 109.5 | 112.3 | 114.0 | 115.2 |
| **4.7** | 99.4 | 100.3 | 101.8 | 104.3 | 107.2 | 110.2 | 113.0 | 114.8 | 115.9 |
| **4.8** | 100.0 | 100.9 | 102.4 | 104.9 | 107.8 | 110.9 | 113.7 | 115.5 | 116.6 |
| **4.9** | 100.6 | 101.5 | 103.0 | 105.6 | 108.5 | 111.6 | 114.4 | 116.2 | 117.4 |
| **5.0** | 101.2 | 102.1 | 103.6 | 106.2 | 109.1 | 112.2 | 115.1 | 116.9 | 118.1 |
| **5.1** | 101.7 | 102.7 | 104.2 | 106.8 | 109.8 | 112.9 | 115.8 | 117.6 | 118.8 |
| **5.2** | 102.3 | 103.3 | 104.8 | 107.4 | 110.5 | 113.6 | 116.5 | 118.3 | 119.5 |
| **5.3** | 102.9 | 103.9 | 105.4 | 108.1 | 111.1 | 114.3 | 117.2 | 119.1 | 120.3 |
| **5.4** | 103.5 | 104.5 | 106.0 | 108.7 | 111.8 | 114.9 | 117.9 | 119.8 | 121.0 |
| **5.5** | 104.1 | 105.1 | 106.6 | 109.3 | 112.4 | 115.6 | 118.6 | 120.5 | 121.7 |
| **5.6** | 104.6 | 105.6 | 107.2 | 109.9 | 113.1 | 116.3 | 119.3 | 121.2 | 122.4 |
| **5.7** | 105.2 | 106.2 | 107.8 | 110.5 | 113.7 | 117.0 | 120.0 | 121.9 | 123.1 |
| **5.8** | 105.8 | 106.8 | 108.4 | 111.2 | 114.3 | 117.6 | 120.7 | 122.6 | 123.8 |
| **5.9** | 106.3 | 107.4 | 109.0 | 111.8 | 115.0 | 118.3 | 121.4 | 123.3 | 124.5 |
| **6.0** | 106.9 | 107.9 | 109.6 | 112.4 | 115.6 | 119.0 | 122.1 | 124.0 | 125.2 |
| **6.1** | 107.5 | 108.5 | 110.2 | 113.0 | 116.3 | 119.6 | 122.8 | 124.7 | 125.9 |
| **6.2** | 108.0 | 109.1 | 110.8 | 113.6 | 116.9 | 120.3 | 123.4 | 125.4 | 126.6 |
| **6.3** | 108.6 | 109.7 | 111.4 | 114.2 | 117.6 | 121.0 | 124.1 | 126.1 | 127.3 |
| **6.4** | 109.1 | 110.2 | 111.9 | 114.9 | 118.2 | 121.6 | 124.8 | 126.7 | 128.0 |
| **6.5** | 109.7 | 110.8 | 112.5 | 115.5 | 118.8 | 122.3 | 125.5 | 127.4 | 128.7 |
| **6.6** | 110.2 | 111.4 | 113.1 | 116.1 | 119.5 | 122.9 | 126.2 | 128.1 | 129.4 |
| **6.7** | 110.8 | 111.9 | 113.7 | 116.7 | 120.1 | 123.6 | 126.8 | 128.8 | 130.1 |
| **6.8** | 111.3 | 112.5 | 114.3 | 117.3 | 120.7 | 124.3 | 127.5 | 129.5 | 130.8 |
| **6.9** | 111.9 | 113.0 | 114.8 | 117.9 | 121.4 | 124.9 | 128.2 | 130.2 | 131.5 |
| **7.0** | 112.4 | 113.6 | 115.4 | 118.5 | 122.0 | 125.6 | 128.8 | 130.8 | 132.1 |
| **7.1** | 113.0 | 114.1 | 116.0 | 119.1 | 122.6 | 126.2 | 129.5 | 131.5 | 132.8 |
| **7.2** | 113.5 | 114.7 | 116.6 | 119.7 | 123.3 | 126.9 | 130.2 | 132.2 | 133.5 |
| **7.3** | 114.0 | 115.2 | 117.1 | 120.3 | 123.9 | 127.5 | 130.9 | 132.9 | 134.2 |
| **7.4** | 114.6 | 115.8 | 117.7 | 120.9 | 124.5 | 128.2 | 131.5 | 133.6 | 134.9 |
| **7.5** | 115.1 | 116.3 | 118.3 | 121.5 | 125.2 | 128.9 | 132.2 | 134.2 | 135.6 |
| **7.6** | 115.6 | 116.9 | 118.8 | 122.1 | 125.8 | 129.5 | 132.9 | 134.9 | 136.2 |
| **7.7** | 116.1 | 117.4 | 119.4 | 122.7 | 126.4 | 130.2 | 133.6 | 135.6 | 136.9 |
| **7.8** | 116.7 | 118.0 | 120.0 | 123.3 | 127.1 | 130.8 | 134.2 | 136.3 | 137.6 |
| **7.9** | 117.2 | 118.5 | 120.5 | 123.9 | 127.7 | 131.5 | 134.9 | 137.0 | 138.3 |
| **8.0** | 117.7 | 119.0 | 121.1 | 124.5 | 128.3 | 132.1 | 135.6 | 137.6 | 139.0 |
| **8.1** | 118.2 | 119.6 | 121.6 | 125.1 | 129.0 | 132.8 | 136.2 | 138.3 | 139.7 |
| **8.2** | 118.7 | 120.1 | 122.2 | 125.7 | 129.6 | 133.4 | 136.9 | 139.0 | 140.3 |
| **8.3** | 119.3 | 120.6 | 122.8 | 126.3 | 130.2 | 134.1 | 137.6 | 139.7 | 141.0 |
| **8.4** | 119.8 | 121.2 | 123.3 | 126.9 | 130.8 | 134.7 | 138.3 | 140.3 | 141.7 |
| **8.5** | 120.3 | 121.7 | 123.9 | 127.5 | 131.5 | 135.4 | 138.9 | 141.0 | 142.4 |
| **8.6** | 120.8 | 122.2 | 124.4 | 128.1 | 132.1 | 136.0 | 139.6 | 141.7 | 143.1 |
| **8.7** | 121.3 | 122.8 | 125.0 | 128.7 | 132.7 | 136.7 | 140.3 | 142.4 | 143.8 |
| **8.8** | 121.8 | 123.3 | 125.5 | 129.2 | 133.3 | 137.3 | 140.9 | 143.1 | 144.4 |
| **8.9** | 122.4 | 123.8 | 126.1 | 129.8 | 133.9 | 138.0 | 141.6 | 143.7 | 145.1 |
| **9.0** | 122.9 | 124.4 | 126.7 | 130.4 | 134.6 | 138.6 | 142.3 | 144.4 | 145.8 |
| **9.1** | 123.4 | 124.9 | 127.2 | 131.0 | 135.2 | 139.3 | 142.9 | 145.1 | 146.5 |
| **9.2** | 124.0 | 125.5 | 127.8 | 131.6 | 135.8 | 139.9 | 143.6 | 145.8 | 147.2 |
| **9.3** | 124.5 | 126.0 | 128.3 | 132.2 | 136.4 | 140.6 | 144.3 | 146.5 | 147.9 |
| **9.4** | 125.0 | 126.6 | 128.9 | 132.8 | 137.0 | 141.2 | 144.9 | 147.1 | 148.6 |
| **9.5** | 125.6 | 127.1 | 129.5 | 133.4 | 137.6 | 141.8 | 145.6 | 147.8 | 149.2 |
| **9.6** | 126.1 | 127.7 | 130.0 | 134.0 | 138.2 | 142.5 | 146.3 | 148.5 | 149.9 |
| **9.7** | 126.7 | 128.2 | 130.6 | 134.5 | 138.9 | 143.1 | 146.9 | 149.2 | 150.6 |
| **9.8** | 127.2 | 128.8 | 131.2 | 135.1 | 139.5 | 143.7 | 147.6 | 149.8 | 151.3 |
| **9.9** | 127.8 | 129.3 | 131.7 | 135.7 | 140.1 | 144.4 | 148.2 | 150.5 | 151.9 |
| **10.0** | 128.3 | 129.9 | 132.3 | 136.3 | 140.7 | 145.0 | 148.8 | 151.1 | 152.6 |
| **10.1** | 128.9 | 130.4 | 132.9 | 136.9 | 141.3 | 145.6 | 149.5 | 151.8 | 153.3 |
| **10.2** | 129.4 | 131.0 | 133.4 | 137.4 | 141.8 | 146.2 | 150.1 | 152.4 | 153.9 |
| **10.3** | 130.0 | 131.6 | 134.0 | 138.0 | 142.4 | 146.8 | 150.7 | 153.1 | 154.6 |
| **10.4** | 130.5 | 132.1 | 134.5 | 138.6 | 143.0 | 147.4 | 151.3 | 153.7 | 155.2 |
| **10.5** | 131.1 | 132.7 | 135.1 | 139.1 | 143.6 | 148.0 | 152.0 | 154.3 | 155.8 |
| **10.6** | 131.6 | 133.2 | 135.7 | 139.7 | 144.2 | 148.6 | 152.6 | 154.9 | 156.4 |
| **10.7** | 132.2 | 133.8 | 136.2 | 140.3 | 144.7 | 149.2 | 153.2 | 155.5 | 157.1 |
| **10.8** | 132.8 | 134.3 | 136.8 | 140.8 | 145.3 | 149.8 | 153.7 | 156.1 | 157.7 |
| **10.9** | 133.3 | 134.9 | 137.3 | 141.4 | 145.9 | 150.3 | 154.3 | 156.7 | 158.3 |
| **11.0** | 133.9 | 135.5 | 137.9 | 141.9 | 146.4 | 150.9 | 154.9 | 157.3 | 158.8 |
| **11.1** | 134.4 | 136.0 | 138.4 | 142.5 | 147.0 | 151.4 | 155.5 | 157.9 | 159.4 |
| **11.2** | 135.0 | 136.6 | 139.0 | 143.0 | 147.5 | 152.0 | 156.0 | 158.4 | 160.0 |
| **11.3** | 135.6 | 137.1 | 139.6 | 143.6 | 148.1 | 152.5 | 156.6 | 159.0 | 160.5 |
| **11.4** | 136.1 | 137.7 | 140.1 | 144.1 | 148.6 | 153.1 | 157.1 | 159.5 | 161.1 |
| **11.5** | 136.7 | 138.2 | 140.6 | 144.7 | 149.1 | 153.6 | 157.6 | 160.1 | 161.6 |
| **11.6** | 137.2 | 138.8 | 141.2 | 145.2 | 149.6 | 154.1 | 158.2 | 160.6 | 162.2 |
| **11.7** | 137.8 | 139.3 | 141.7 | 145.7 | 150.1 | 154.6 | 158.7 | 161.1 | 162.7 |
| **11.8** | 138.3 | 139.9 | 142.2 | 146.2 | 150.6 | 155.1 | 159.2 | 161.6 | 163.2 |
| **11.9** | 138.9 | 140.4 | 142.8 | 146.7 | 151.1 | 155.6 | 159.6 | 162.1 | 163.7 |
| **12.0** | 139.4 | 140.9 | 143.3 | 147.2 | 151.6 | 156.1 | 160.1 | 162.5 | 164.1 |
| **12.1** | 139.9 | 141.4 | 143.8 | 147.7 | 152.1 | 156.5 | 160.6 | 163.0 | 164.6 |
| **12.2** | 140.4 | 141.9 | 144.3 | 148.2 | 152.5 | 157.0 | 161.0 | 163.4 | 165.0 |
| **12.3** | 141.0 | 142.4 | 144.7 | 148.6 | 153.0 | 157.4 | 161.4 | 163.9 | 165.5 |
| **12.4** | 141.5 | 142.9 | 145.2 | 149.1 | 153.4 | 157.8 | 161.9 | 164.3 | 165.9 |
| **12.5** | 141.9 | 143.4 | 145.7 | 149.5 | 153.9 | 158.3 | 162.3 | 164.7 | 166.3 |
| **12.6** | 142.4 | 143.9 | 146.1 | 150.0 | 154.3 | 158.7 | 162.7 | 165.1 | 166.7 |
| **12.7** | 142.9 | 144.3 | 146.6 | 150.4 | 154.7 | 159.0 | 163.0 | 165.5 | 167.1 |
| **12.8** | 143.4 | 144.8 | 147.0 | 150.8 | 155.1 | 159.4 | 163.4 | 165.8 | 167.4 |
| **12.9** | 143.8 | 145.2 | 147.4 | 151.2 | 155.4 | 159.8 | 163.8 | 166.2 | 167.8 |
| **13.0** | 144.3 | 145.7 | 147.9 | 151.6 | 155.8 | 160.1 | 164.1 | 166.5 | 168.1 |
| **13.1** | 144.7 | 146.1 | 148.3 | 152.0 | 156.2 | 160.5 | 164.4 | 166.9 | 168.4 |
| **13.2** | 145.1 | 146.5 | 148.7 | 152.3 | 156.5 | 160.8 | 164.8 | 167.2 | 168.7 |
| **13.3** | 145.5 | 146.9 | 149.0 | 152.7 | 156.9 | 161.1 | 165.1 | 167.5 | 169.0 |
| **13.4** | 145.9 | 147.3 | 149.4 | 153.0 | 157.2 | 161.4 | 165.4 | 167.8 | 169.3 |
| **13.5** | 146.3 | 147.6 | 149.8 | 153.4 | 157.5 | 161.7 | 165.7 | 168.0 | 169.6 |
| **13.6** | 146.6 | 148.0 | 150.1 | 153.7 | 157.8 | 162.0 | 165.9 | 168.3 | 169.9 |
| **13.7** | 147.0 | 148.3 | 150.4 | 154.0 | 158.1 | 162.3 | 166.2 | 168.6 | 170.1 |
| **13.8** | 147.3 | 148.7 | 150.8 | 154.3 | 158.4 | 162.6 | 166.4 | 168.8 | 170.4 |
| **13.9** | 147.7 | 149.0 | 151.1 | 154.6 | 158.6 | 162.8 | 166.7 | 169.1 | 170.6 |
| **14.0** | 148.0 | 149.3 | 151.4 | 154.9 | 158.9 | 163.1 | 166.9 | 169.3 | 170.8 |
| **14.1** | 148.3 | 149.6 | 151.7 | 155.2 | 159.2 | 163.3 | 167.1 | 169.5 | 171.0 |
| **14.2** | 148.6 | 149.9 | 151.9 | 155.4 | 159.4 | 163.5 | 167.4 | 169.7 | 171.2 |
| **14.3** | 148.9 | 150.2 | 152.2 | 155.7 | 159.6 | 163.7 | 167.6 | 169.9 | 171.4 |
| **14.4** | 149.1 | 150.4 | 152.4 | 155.9 | 159.9 | 164.0 | 167.8 | 170.1 | 171.6 |
| **14.5** | 149.4 | 150.7 | 152.7 | 156.1 | 160.1 | 164.2 | 167.9 | 170.3 | 171.8 |
| **14.6** | 149.6 | 150.9 | 152.9 | 156.3 | 160.3 | 164.3 | 168.1 | 170.4 | 172.0 |
| **14.7** | 149.9 | 151.1 | 153.1 | 156.6 | 160.5 | 164.5 | 168.3 | 170.6 | 172.1 |
| **14.8** | 150.1 | 151.4 | 153.3 | 156.8 | 160.7 | 164.7 | 168.5 | 170.8 | 172.3 |
| **14.9** | 150.3 | 151.6 | 153.5 | 156.9 | 160.8 | 164.9 | 168.6 | 170.9 | 172.4 |
| **15.0** | 150.5 | 151.8 | 153.7 | 157.1 | 161.0 | 165.0 | 168.8 | 171.1 | 172.6 |
| **15.1** | 150.7 | 151.9 | 153.9 | 157.3 | 161.2 | 165.2 | 168.9 | 171.2 | 172.7 |
| **15.2** | 150.9 | 152.1 | 154.1 | 157.5 | 161.3 | 165.3 | 169.0 | 171.3 | 172.8 |
| **15.3** | 151.0 | 152.3 | 154.2 | 157.6 | 161.5 | 165.5 | 169.2 | 171.4 | 172.9 |
| **15.4** | 151.2 | 152.4 | 154.4 | 157.8 | 161.6 | 165.6 | 169.3 | 171.6 | 173.0 |
| **15.5** | 151.3 | 152.6 | 154.5 | 157.9 | 161.7 | 165.7 | 169.4 | 171.7 | 173.2 |
| **15.6** | 151.5 | 152.7 | 154.7 | 158.0 | 161.9 | 165.8 | 169.5 | 171.8 | 173.3 |
| **15.7** | 151.6 | 152.9 | 154.8 | 158.2 | 162.0 | 165.9 | 169.6 | 171.9 | 173.4 |
| **15.8** | 151.7 | 153.0 | 154.9 | 158.3 | 162.1 | 166.0 | 169.7 | 172.0 | 173.4 |
| **15.9** | 151.9 | 153.1 | 155.1 | 158.4 | 162.2 | 166.1 | 169.8 | 172.1 | 173.5 |
| **16.0** | 152.0 | 153.2 | 155.2 | 158.5 | 162.3 | 166.2 | 169.9 | 172.1 | 173.6 |
| **16.1** | 152.1 | 153.3 | 155.3 | 158.6 | 162.4 | 166.3 | 170.0 | 172.2 | 173.7 |
| **16.2** | 152.2 | 153.4 | 155.4 | 158.7 | 162.5 | 166.4 | 170.1 | 172.3 | 173.8 |
| **16.3** | 152.3 | 153.5 | 155.5 | 158.8 | 162.6 | 166.5 | 170.2 | 172.4 | 173.9 |
| **16.4** | 152.4 | 153.6 | 155.6 | 158.9 | 162.7 | 166.6 | 170.2 | 172.5 | 173.9 |
| **16.5** | 152.4 | 153.7 | 155.6 | 159.0 | 162.8 | 166.7 | 170.3 | 172.5 | 174.0 |
| **16.6** | 152.5 | 153.8 | 155.7 | 159.0 | 162.8 | 166.7 | 170.4 | 172.6 | 174.1 |
| **16.7** | 152.6 | 153.8 | 155.8 | 159.1 | 162.9 | 166.8 | 170.4 | 172.7 | 174.1 |
| **16.8** | 152.7 | 153.9 | 155.9 | 159.2 | 163.0 | 166.9 | 170.5 | 172.7 | 174.2 |
| **16.9** | 152.7 | 154.0 | 155.9 | 159.2 | 163.0 | 166.9 | 170.6 | 172.8 | 174.2 |
| **17.0** | 152.8 | 154.0 | 156.0 | 159.3 | 163.1 | 167.0 | 170.6 | 172.8 | 174.3 |
| **17.1** | 152.9 | 154.1 | 156.0 | 159.4 | 163.2 | 167.1 | 170.7 | 172.9 | 174.4 |
| **17.2** | 152.9 | 154.2 | 156.1 | 159.4 | 163.2 | 167.1 | 170.7 | 173.0 | 174.4 |
| **17.3** | 153.0 | 154.2 | 156.1 | 159.5 | 163.3 | 167.2 | 170.8 | 173.0 | 174.5 |
| **17.4** | 153.0 | 154.2 | 156.2 | 159.5 | 163.3 | 167.2 | 170.8 | 173.1 | 174.5 |
| **17.5** | 153.0 | 154.3 | 156.2 | 159.6 | 163.4 | 167.3 | 170.9 | 173.1 | 174.6 |
| **17.6** | 153.1 | 154.3 | 156.3 | 159.6 | 163.4 | 167.3 | 170.9 | 173.2 | 174.6 |
| **17.7** | 153.1 | 154.4 | 156.3 | 159.6 | 163.4 | 167.4 | 171.0 | 173.2 | 174.7 |
| **17.8** | 153.1 | 154.4 | 156.3 | 159.7 | 163.5 | 167.4 | 171.0 | 173.3 | 174.7 |
| **17.9** | 153.2 | 154.4 | 156.4 | 159.7 | 163.5 | 167.4 | 171.1 | 173.3 | 174.8 |
| **18.0** | 153.2 | 154.5 | 156.4 | 159.7 | 163.5 | 167.5 | 171.1 | 173.3 | 174.8 |
| **18.1** | 153.2 | 154.5 | 156.4 | 159.8 | 163.6 | 167.5 | 171.1 | 173.4 | 174.9 |
| **18.2** | 153.2 | 154.5 | 156.4 | 159.8 | 163.6 | 167.5 | 171.2 | 173.4 | 174.9 |
| **18.3** | 153.3 | 154.5 | 156.5 | 159.8 | 163.6 | 167.6 | 171.2 | 173.5 | 174.9 |
| **18.4** | 153.3 | 154.5 | 156.5 | 159.8 | 163.6 | 167.6 | 171.3 | 173.5 | 175.0 |
| **18.5** | 153.3 | 154.6 | 156.5 | 159.8 | 163.7 | 167.6 | 171.3 | 173.5 | 175.0 |
| **18.6** | 153.3 | 154.6 | 156.5 | 159.8 | 163.7 | 167.6 | 171.3 | 173.6 | 175.1 |
| **18.7** | 153.3 | 154.6 | 156.5 | 159.9 | 163.7 | 167.6 | 171.3 | 173.6 | 175.1 |
| **18.8** | 153.3 | 154.6 | 156.5 | 159.9 | 163.7 | 167.7 | 171.4 | 173.6 | 175.1 |
| **18.9** | 153.3 | 154.6 | 156.5 | 159.9 | 163.7 | 167.7 | 171.4 | 173.6 | 175.1 |
| **19.0** | 153.3 | 154.6 | 156.5 | 159.9 | 163.7 | 167.7 | 171.4 | 173.7 | 175.2 |
| **19.1** | 153.3 | 154.6 | 156.5 | 159.9 | 163.7 | 167.7 | 171.4 | 173.7 | 175.2 |
| **19.2** | 153.3 | 154.6 | 156.5 | 159.9 | 163.7 | 167.7 | 171.4 | 173.7 | 175.2 |
| **19.3** | 153.3 | 154.6 | 156.5 | 159.9 | 163.7 | 167.7 | 171.4 | 173.7 | 175.2 |
| **19.4** | 153.3 | 154.6 | 156.5 | 159.9 | 163.7 | 167.7 | 171.4 | 173.7 | 175.2 |
| **19.5** | 153.3 | 154.6 | 156.5 | 159.9 | 163.7 | 167.7 | 171.4 | 173.7 | 175.2 |
| **19.6** | 153.3 | 154.6 | 156.5 | 159.8 | 163.7 | 167.7 | 171.4 | 173.7 | 175.3 |
| **19.7** | 153.3 | 154.6 | 156.5 | 159.8 | 163.7 | 167.7 | 171.4 | 173.8 | 175.3 |
| **19.8** | 153.3 | 154.6 | 156.5 | 159.8 | 163.7 | 167.7 | 171.5 | 173.8 | 175.3 |
| **19.9** | 153.3 | 154.6 | 156.5 | 159.8 | 163.7 | 167.7 | 171.5 | 173.8 | 175.3 |
| **20.0** | 153.3 | 154.6 | 156.5 | 159.9 | 163.7 | 167.7 | 171.5 | 173.8 | 175.4 |

**TABLE B. Men's stature**

| **Stature percentiles for men** | | | | | | | | | |
| --- | --- | --- | --- | --- | --- | --- | --- | --- | --- |
| **Age** | **P3** | **P5** | **P10** | **P25** | **P50** | **P75** | **P90** | **P95** | **P97** |
| **3.0** | 90.8 | 91.5 | 92.7 | 94.7 | 97.1 | 99.6 | 102.0 | 103.5 | 104.5 |
| **3.1** | 91.4 | 92.1 | 93.3 | 95.4 | 97.8 | 100.3 | 102.7 | 104.2 | 105.2 |
| **3.2** | 92.0 | 92.7 | 93.9 | 96.0 | 98.5 | 101.0 | 103.4 | 104.9 | 105.9 |
| **3.3** | 92.5 | 93.3 | 94.5 | 96.7 | 99.1 | 101.7 | 104.2 | 105.7 | 106.7 |
| **3.4** | 93.1 | 93.9 | 95.2 | 97.3 | 99.8 | 102.4 | 104.9 | 106.4 | 107.4 |
| **3.5** | 93.7 | 94.5 | 95.8 | 97.9 | 100.5 | 103.1 | 105.6 | 107.1 | 108.1 |
| **3.6** | 94.3 | 95.1 | 96.4 | 98.6 | 101.1 | 103.8 | 106.3 | 107.8 | 108.9 |
| **3.7** | 94.8 | 95.7 | 97.0 | 99.2 | 101.8 | 104.5 | 107.0 | 108.6 | 109.6 |
| **3.8** | 95.4 | 96.3 | 97.6 | 99.9 | 102.5 | 105.2 | 107.7 | 109.3 | 110.3 |
| **3.9** | 96.0 | 96.9 | 98.2 | 100.5 | 103.1 | 105.9 | 108.4 | 110.0 | 111.0 |
| **4.0** | 96.6 | 97.4 | 98.8 | 101.1 | 103.8 | 106.6 | 109.2 | 110.7 | 111.8 |
| **4.1** | 97.1 | 98.0 | 99.4 | 101.8 | 104.5 | 107.3 | 109.9 | 111.5 | 112.5 |
| **4.2** | 97.7 | 98.6 | 100.0 | 102.4 | 105.2 | 108.0 | 110.6 | 112.2 | 113.2 |
| **4.3** | 98.3 | 99.2 | 100.6 | 103.1 | 105.8 | 108.7 | 111.3 | 112.9 | 114.0 |
| **4.4** | 98.9 | 99.8 | 101.2 | 103.7 | 106.5 | 109.4 | 112.0 | 113.6 | 114.7 |
| **4.5** | 99.4 | 100.4 | 101.9 | 104.4 | 107.2 | 110.1 | 112.8 | 114.4 | 115.4 |
| **4.6** | 100.0 | 101.0 | 102.5 | 105.0 | 107.9 | 110.8 | 113.5 | 115.1 | 116.2 |
| **4.7** | 100.6 | 101.6 | 103.1 | 105.6 | 108.5 | 111.5 | 114.2 | 115.8 | 116.9 |
| **4.8** | 101.2 | 102.1 | 103.7 | 106.3 | 109.2 | 112.2 | 114.9 | 116.6 | 117.6 |
| **4.9** | 101.7 | 102.7 | 104.3 | 106.9 | 109.9 | 112.9 | 115.6 | 117.3 | 118.4 |
| **5.0** | 102.3 | 103.3 | 104.9 | 107.5 | 110.5 | 113.6 | 116.3 | 118.0 | 119.1 |
| **5.1** | 102.9 | 103.9 | 105.5 | 108.2 | 111.2 | 114.3 | 117.0 | 118.7 | 119.8 |
| **5.2** | 103.4 | 104.5 | 106.1 | 108.8 | 111.9 | 114.9 | 117.7 | 119.4 | 120.5 |
| **5.3** | 104.0 | 105.0 | 106.7 | 109.4 | 112.5 | 115.6 | 118.4 | 120.1 | 121.3 |
| **5.4** | 104.5 | 105.6 | 107.3 | 110.1 | 113.2 | 116.3 | 119.2 | 120.9 | 122.0 |
| **5.5** | 105.1 | 106.2 | 107.9 | 110.7 | 113.8 | 117.0 | 119.9 | 121.6 | 122.7 |
| **5.6** | 105.7 | 106.8 | 108.4 | 111.3 | 114.5 | 117.7 | 120.6 | 122.3 | 123.4 |
| **5.7** | 106.2 | 107.3 | 109.0 | 111.9 | 115.1 | 118.3 | 121.2 | 123.0 | 124.1 |
| **5.8** | 106.8 | 107.9 | 109.6 | 112.5 | 115.8 | 119.0 | 121.9 | 123.7 | 124.8 |
| **5.9** | 107.3 | 108.5 | 110.2 | 113.1 | 116.4 | 119.7 | 122.6 | 124.4 | 125.5 |
| **6.0** | 107.9 | 109.0 | 110.8 | 113.7 | 117.0 | 120.3 | 123.3 | 125.1 | 126.2 |
| **6.1** | 108.4 | 109.6 | 111.4 | 114.4 | 117.7 | 121.0 | 124.0 | 125.8 | 127.0 |
| **6.2** | 109.0 | 110.1 | 111.9 | 115.0 | 118.3 | 121.7 | 124.7 | 126.5 | 127.7 |
| **6.3** | 109.5 | 110.7 | 112.5 | 115.5 | 118.9 | 122.3 | 125.3 | 127.2 | 128.3 |
| **6.4** | 110.0 | 111.2 | 113.1 | 116.1 | 119.6 | 123.0 | 126.0 | 127.9 | 129.0 |
| **6.5** | 110.6 | 111.8 | 113.6 | 116.7 | 120.2 | 123.6 | 126.7 | 128.5 | 129.7 |
| **6.6** | 111.1 | 112.3 | 114.2 | 117.3 | 120.8 | 124.2 | 127.4 | 129.2 | 130.4 |
| **6.7** | 111.6 | 112.9 | 114.8 | 117.9 | 121.4 | 124.9 | 128.0 | 129.9 | 131.1 |
| **6.8** | 112.2 | 113.4 | 115.3 | 118.5 | 122.0 | 125.5 | 128.7 | 130.6 | 131.8 |
| **6.9** | 112.7 | 114.0 | 115.9 | 119.1 | 122.6 | 126.1 | 129.3 | 131.2 | 132.5 |
| **7.0** | 113.2 | 114.5 | 116.4 | 119.6 | 123.2 | 126.8 | 130.0 | 131.9 | 133.1 |
| **7.1** | 113.8 | 115.0 | 117.0 | 120.2 | 123.8 | 127.4 | 130.6 | 132.5 | 133.8 |
| **7.2** | 114.3 | 115.6 | 117.5 | 120.8 | 124.4 | 128.0 | 131.3 | 133.2 | 134.4 |
| **7.3** | 114.8 | 116.1 | 118.1 | 121.4 | 125.0 | 128.6 | 131.9 | 133.8 | 135.1 |
| **7.4** | 115.3 | 116.6 | 118.6 | 121.9 | 125.6 | 129.2 | 132.5 | 134.5 | 135.8 |
| **7.5** | 115.8 | 117.1 | 119.1 | 122.5 | 126.2 | 129.9 | 133.2 | 135.1 | 136.4 |
| **7.6** | 116.4 | 117.7 | 119.7 | 123.0 | 126.8 | 130.5 | 133.8 | 135.8 | 137.1 |
| **7.7** | 116.9 | 118.2 | 120.2 | 123.6 | 127.3 | 131.1 | 134.4 | 136.4 | 137.7 |
| **7.8** | 117.4 | 118.7 | 120.7 | 124.1 | 127.9 | 131.7 | 135.0 | 137.0 | 138.3 |
| **7.9** | 117.9 | 119.2 | 121.3 | 124.7 | 128.5 | 132.3 | 135.6 | 137.7 | 139.0 |
| **8.0** | 118.4 | 119.7 | 121.8 | 125.2 | 129.1 | 132.9 | 136.3 | 138.3 | 139.6 |
| **8.1** | 118.9 | 120.2 | 122.3 | 125.8 | 129.6 | 133.5 | 136.9 | 138.9 | 140.3 |
| **8.2** | 119.4 | 120.7 | 122.8 | 126.3 | 130.2 | 134.0 | 137.5 | 139.6 | 140.9 |
| **8.3** | 119.9 | 121.2 | 123.4 | 126.9 | 130.8 | 134.6 | 138.1 | 140.2 | 141.5 |
| **8.4** | 120.4 | 121.7 | 123.9 | 127.4 | 131.3 | 135.2 | 138.7 | 140.8 | 142.1 |
| **8.5** | 120.9 | 122.2 | 124.4 | 128.0 | 131.9 | 135.8 | 139.3 | 141.4 | 142.8 |
| **8.6** | 121.3 | 122.7 | 124.9 | 128.5 | 132.5 | 136.4 | 139.9 | 142.0 | 143.4 |
| **8.7** | 121.8 | 123.2 | 125.4 | 129.0 | 133.0 | 137.0 | 140.5 | 142.7 | 144.0 |
| **8.8** | 122.3 | 123.7 | 125.9 | 129.6 | 133.6 | 137.6 | 141.2 | 143.3 | 144.7 |
| **8.9** | 122.8 | 124.2 | 126.4 | 130.1 | 134.1 | 138.2 | 141.8 | 143.9 | 145.3 |
| **9.0** | 123.3 | 124.7 | 126.9 | 130.6 | 134.7 | 138.8 | 142.4 | 144.5 | 145.9 |
| **9.1** | 123.7 | 125.2 | 127.4 | 131.2 | 135.3 | 139.3 | 143.0 | 145.2 | 146.6 |
| **9.2** | 124.2 | 125.7 | 127.9 | 131.7 | 135.8 | 139.9 | 143.6 | 145.8 | 147.2 |
| **9.3** | 124.7 | 126.2 | 128.4 | 132.2 | 136.4 | 140.5 | 144.2 | 146.4 | 147.8 |
| **9.4** | 125.1 | 126.6 | 128.9 | 132.7 | 136.9 | 141.1 | 144.8 | 147.0 | 148.5 |
| **9.5** | 125.6 | 127.1 | 129.4 | 133.3 | 137.5 | 141.7 | 145.4 | 147.7 | 149.1 |
| **9.6** | 126.1 | 127.6 | 129.9 | 133.8 | 138.1 | 142.3 | 146.1 | 148.3 | 149.8 |
| **9.7** | 126.5 | 128.1 | 130.4 | 134.3 | 138.6 | 142.9 | 146.7 | 148.9 | 150.4 |
| **9.8** | 127.0 | 128.6 | 130.9 | 134.9 | 139.2 | 143.5 | 147.3 | 149.6 | 151.1 |
| **9.9** | 127.5 | 129.0 | 131.4 | 135.4 | 139.8 | 144.1 | 147.9 | 150.2 | 151.7 |
| **10.0** | 127.9 | 129.5 | 131.9 | 135.9 | 140.3 | 144.7 | 148.6 | 150.9 | 152.4 |
| **10.1** | 128.4 | 130.0 | 132.4 | 136.4 | 140.9 | 145.3 | 149.2 | 151.5 | 153.0 |
| **10.2** | 128.9 | 130.5 | 132.9 | 137.0 | 141.4 | 145.9 | 149.8 | 152.2 | 153.7 |
| **10.3** | 129.3 | 131.0 | 133.4 | 137.5 | 142.0 | 146.5 | 150.5 | 152.8 | 154.4 |
| **10.4** | 129.8 | 131.4 | 133.9 | 138.1 | 142.6 | 147.1 | 151.1 | 153.5 | 155.0 |
| **10.5** | 130.3 | 131.9 | 134.4 | 138.6 | 143.2 | 147.7 | 151.7 | 154.1 | 155.7 |
| **10.6** | 130.8 | 132.4 | 134.9 | 139.1 | 143.7 | 148.3 | 152.4 | 154.8 | 156.4 |
| **10.7** | 131.2 | 132.9 | 135.5 | 139.7 | 144.3 | 148.9 | 153.0 | 155.5 | 157.0 |
| **10.8** | 131.7 | 133.4 | 136.0 | 140.2 | 144.9 | 149.5 | 153.7 | 156.1 | 157.7 |
| **10.9** | 132.2 | 133.9 | 136.5 | 140.8 | 145.5 | 150.2 | 154.3 | 156.8 | 158.4 |
| **11.0** | 132.7 | 134.4 | 137.0 | 141.3 | 146.1 | 150.8 | 155.0 | 157.5 | 159.1 |
| **11.1** | 133.2 | 134.9 | 137.5 | 141.9 | 146.7 | 151.4 | 155.6 | 158.1 | 159.8 |
| **11.2** | 133.7 | 135.4 | 138.1 | 142.4 | 147.2 | 152.0 | 156.3 | 158.8 | 160.5 |
| **11.3** | 134.2 | 135.9 | 138.6 | 143.0 | 147.8 | 152.7 | 156.9 | 159.5 | 161.1 |
| **11.4** | 134.7 | 136.5 | 139.1 | 143.6 | 148.4 | 153.3 | 157.6 | 160.2 | 161.8 |
| **11.5** | 135.2 | 137.0 | 139.7 | 144.1 | 149.0 | 153.9 | 158.3 | 160.8 | 162.5 |
| **11.6** | 135.7 | 137.5 | 140.2 | 144.7 | 149.6 | 154.5 | 158.9 | 161.5 | 163.2 |
| **11.7** | 136.3 | 138.0 | 140.8 | 145.3 | 150.2 | 155.2 | 159.6 | 162.2 | 163.9 |
| **11.8** | 136.8 | 138.6 | 141.3 | 145.9 | 150.8 | 155.8 | 160.2 | 162.9 | 164.6 |
| **11.9** | 137.3 | 139.1 | 141.9 | 146.4 | 151.5 | 156.4 | 160.9 | 163.5 | 165.3 |
| **12.0** | 137.9 | 139.7 | 142.4 | 147.0 | 152.1 | 157.1 | 161.5 | 164.2 | 165.9 |
| **12.1** | 138.4 | 140.2 | 143.0 | 147.6 | 152.7 | 157.7 | 162.2 | 164.9 | 166.6 |
| **12.2** | 139.0 | 140.8 | 143.6 | 148.2 | 153.3 | 158.3 | 162.9 | 165.5 | 167.3 |
| **12.3** | 139.5 | 141.3 | 144.1 | 148.8 | 153.9 | 159.0 | 163.5 | 166.2 | 167.9 |
| **12.4** | 140.1 | 141.9 | 144.7 | 149.4 | 154.5 | 159.6 | 164.1 | 166.9 | 168.6 |
| **12.5** | 140.6 | 142.5 | 145.3 | 150.0 | 155.1 | 160.2 | 164.8 | 167.5 | 169.3 |
| **12.6** | 141.2 | 143.0 | 145.9 | 150.6 | 155.7 | 160.9 | 165.4 | 168.1 | 169.9 |
| **12.7** | 141.8 | 143.6 | 146.5 | 151.2 | 156.4 | 161.5 | 166.1 | 168.8 | 170.5 |
| **12.8** | 142.3 | 144.2 | 147.0 | 151.8 | 157.0 | 162.1 | 166.7 | 169.4 | 171.2 |
| **12.9** | 142.9 | 144.8 | 147.6 | 152.4 | 157.6 | 162.7 | 167.3 | 170.0 | 171.8 |
| **13.0** | 143.5 | 145.4 | 148.2 | 153.0 | 158.2 | 163.3 | 167.9 | 170.6 | 172.4 |
| **13.1** | 144.1 | 145.9 | 148.8 | 153.6 | 158.8 | 163.9 | 168.5 | 171.2 | 173.0 |
| **13.2** | 144.6 | 146.5 | 149.4 | 154.2 | 159.4 | 164.5 | 169.1 | 171.8 | 173.6 |
| **13.3** | 145.2 | 147.1 | 150.0 | 154.7 | 160.0 | 165.1 | 169.7 | 172.4 | 174.2 |
| **13.4** | 145.8 | 147.7 | 150.6 | 155.3 | 160.6 | 165.7 | 170.3 | 173.0 | 174.7 |
| **13.5** | 146.4 | 148.3 | 151.2 | 155.9 | 161.1 | 166.3 | 170.8 | 173.5 | 175.3 |
| **13.6** | 147.0 | 148.9 | 151.8 | 156.5 | 161.7 | 166.8 | 171.4 | 174.1 | 175.8 |
| **13.7** | 147.6 | 149.5 | 152.3 | 157.1 | 162.3 | 167.4 | 171.9 | 174.6 | 176.3 |
| **13.8** | 148.1 | 150.0 | 152.9 | 157.7 | 162.9 | 168.0 | 172.5 | 175.1 | 176.9 |
| **13.9** | 148.7 | 150.6 | 153.5 | 158.2 | 163.4 | 168.5 | 173.0 | 175.7 | 177.4 |
| **14.0** | 149.3 | 151.2 | 154.1 | 158.8 | 164.0 | 169.0 | 173.5 | 176.2 | 177.9 |
| **14.1** | 149.9 | 151.8 | 154.6 | 159.3 | 164.5 | 169.5 | 174.0 | 176.6 | 178.3 |
| **14.2** | 150.4 | 152.3 | 155.2 | 159.9 | 165.0 | 170.0 | 174.5 | 177.1 | 178.8 |
| **14.3** | 151.0 | 152.9 | 155.7 | 160.4 | 165.5 | 170.5 | 175.0 | 177.6 | 179.3 |
| **14.4** | 151.6 | 153.4 | 156.3 | 161.0 | 166.0 | 171.0 | 175.4 | 178.0 | 179.7 |
| **14.5** | 152.1 | 154.0 | 156.8 | 161.5 | 166.5 | 171.5 | 175.9 | 178.5 | 180.1 |
| **14.6** | 152.7 | 154.5 | 157.3 | 162.0 | 167.0 | 172.0 | 176.3 | 178.9 | 180.5 |
| **14.7** | 153.2 | 155.0 | 157.8 | 162.5 | 167.5 | 172.4 | 176.7 | 179.3 | 180.9 |
| **14.8** | 153.7 | 155.5 | 158.3 | 162.9 | 167.9 | 172.8 | 177.1 | 179.7 | 181.3 |
| **14.9** | 154.2 | 156.0 | 158.8 | 163.4 | 168.4 | 173.2 | 177.5 | 180.1 | 181.7 |
| **15.0** | 154.7 | 156.5 | 159.3 | 163.9 | 168.8 | 173.6 | 177.9 | 180.4 | 182.0 |
| **15.1** | 155.2 | 157.0 | 159.8 | 164.3 | 169.2 | 174.0 | 178.3 | 180.8 | 182.4 |
| **15.2** | 155.7 | 157.5 | 160.2 | 164.7 | 169.6 | 174.4 | 178.6 | 181.1 | 182.7 |
| **15.3** | 156.1 | 157.9 | 160.6 | 165.1 | 170.0 | 174.8 | 179.0 | 181.4 | 183.0 |
| **15.4** | 156.5 | 158.3 | 161.1 | 165.5 | 170.4 | 175.1 | 179.3 | 181.7 | 183.3 |
| **15.5** | 157.0 | 158.8 | 161.5 | 165.9 | 170.7 | 175.4 | 179.6 | 182.0 | 183.6 |
| **15.6** | 157.4 | 159.2 | 161.8 | 166.3 | 171.1 | 175.8 | 179.9 | 182.3 | 183.9 |
| **15.7** | 157.8 | 159.5 | 162.2 | 166.6 | 171.4 | 176.1 | 180.2 | 182.6 | 184.2 |
| **15.8** | 158.2 | 159.9 | 162.6 | 167.0 | 171.7 | 176.3 | 180.4 | 182.9 | 184.4 |
| **15.9** | 158.5 | 160.3 | 162.9 | 167.3 | 172.0 | 176.6 | 180.7 | 183.1 | 184.7 |
| **16.0** | 158.9 | 160.6 | 163.3 | 167.6 | 172.3 | 176.9 | 181.0 | 183.4 | 184.9 |
| **16.1** | 159.2 | 160.9 | 163.6 | 167.9 | 172.6 | 177.1 | 181.2 | 183.6 | 185.1 |
| **16.2** | 159.6 | 161.3 | 163.9 | 168.1 | 172.8 | 177.4 | 181.4 | 183.8 | 185.3 |
| **16.3** | 159.9 | 161.6 | 164.2 | 168.4 | 173.1 | 177.6 | 181.6 | 184.0 | 185.6 |
| **16.4** | 160.2 | 161.9 | 164.4 | 168.7 | 173.3 | 177.8 | 181.8 | 184.2 | 185.7 |
| **16.5** | 160.5 | 162.1 | 164.7 | 168.9 | 173.5 | 178.0 | 182.0 | 184.4 | 185.9 |
| **16.6** | 160.7 | 162.4 | 164.9 | 169.1 | 173.7 | 178.2 | 182.2 | 184.6 | 186.1 |
| **16.7** | 161.0 | 162.6 | 165.2 | 169.3 | 173.9 | 178.4 | 182.4 | 184.8 | 186.3 |
| **16.8** | 161.2 | 162.9 | 165.4 | 169.5 | 174.1 | 178.6 | 182.6 | 184.9 | 186.5 |
| **16.9** | 161.5 | 163.1 | 165.6 | 169.7 | 174.3 | 178.7 | 182.7 | 185.1 | 186.6 |
| **17.0** | 161.7 | 163.3 | 165.8 | 169.9 | 174.4 | 178.9 | 182.9 | 185.2 | 186.8 |
| **17.1** | 161.9 | 163.5 | 166.0 | 170.1 | 174.6 | 179.0 | 183.0 | 185.4 | 186.9 |
| **17.2** | 162.1 | 163.7 | 166.1 | 170.2 | 174.7 | 179.2 | 183.2 | 185.5 | 187.1 |
| **17.3** | 162.3 | 163.9 | 166.3 | 170.4 | 174.9 | 179.3 | 183.3 | 185.7 | 187.2 |
| **17.4** | 162.4 | 164.0 | 166.5 | 170.5 | 175.0 | 179.4 | 183.4 | 185.8 | 187.3 |
| **17.5** | 162.6 | 164.2 | 166.6 | 170.6 | 175.1 | 179.5 | 183.5 | 185.9 | 187.4 |
| **17.6** | 162.7 | 164.3 | 166.7 | 170.8 | 175.2 | 179.7 | 183.6 | 186.0 | 187.6 |
| **17.7** | 162.9 | 164.4 | 166.8 | 170.9 | 175.3 | 179.8 | 183.7 | 186.1 | 187.7 |
| **17.8** | 163.0 | 164.6 | 167.0 | 171.0 | 175.4 | 179.9 | 183.8 | 186.2 | 187.8 |
| **17.9** | 163.1 | 164.7 | 167.1 | 171.1 | 175.5 | 179.9 | 183.9 | 186.3 | 187.9 |
| **18.0** | 163.3 | 164.8 | 167.2 | 171.2 | 175.6 | 180.0 | 184.0 | 186.4 | 188.0 |
| **18.1** | 163.4 | 164.9 | 167.3 | 171.3 | 175.7 | 180.1 | 184.1 | 186.5 | 188.1 |
| **18.2** | 163.5 | 165.0 | 167.4 | 171.3 | 175.8 | 180.2 | 184.2 | 186.6 | 188.2 |
| **18.3** | 163.6 | 165.1 | 167.5 | 171.4 | 175.8 | 180.3 | 184.3 | 186.7 | 188.3 |
| **18.4** | 163.7 | 165.2 | 167.5 | 171.5 | 175.9 | 180.4 | 184.4 | 186.8 | 188.4 |
| **18.5** | 163.8 | 165.3 | 167.6 | 171.6 | 176.0 | 180.4 | 184.5 | 186.9 | 188.5 |
| **18.6** | 163.9 | 165.4 | 167.7 | 171.6 | 176.1 | 180.5 | 184.6 | 187.0 | 188.6 |
| **18.7** | 163.9 | 165.4 | 167.8 | 171.7 | 176.1 | 180.6 | 184.7 | 187.1 | 188.7 |
| **18.8** | 164.0 | 165.5 | 167.9 | 171.8 | 176.2 | 180.7 | 184.7 | 187.2 | 188.8 |
| **18.9** | 164.1 | 165.6 | 167.9 | 171.8 | 176.2 | 180.7 | 184.8 | 187.3 | 188.9 |
| **19.0** | 164.2 | 165.7 | 168.0 | 171.9 | 176.3 | 180.8 | 184.9 | 187.4 | 189.0 |
| **19.1** | 164.3 | 165.7 | 168.0 | 171.9 | 176.4 | 180.9 | 185.0 | 187.5 | 189.1 |
| **19.2** | 164.3 | 165.8 | 168.1 | 172.0 | 176.4 | 180.9 | 185.0 | 187.5 | 189.2 |
| **19.3** | 164.4 | 165.9 | 168.2 | 172.1 | 176.5 | 181.0 | 185.1 | 187.6 | 189.3 |
| **19.4** | 164.5 | 165.9 | 168.2 | 172.1 | 176.5 | 181.0 | 185.2 | 187.7 | 189.4 |
| **19.5** | 164.5 | 166.0 | 168.3 | 172.1 | 176.6 | 181.1 | 185.3 | 187.8 | 189.5 |
| **19.6** | 164.6 | 166.1 | 168.3 | 172.2 | 176.6 | 181.1 | 185.3 | 187.9 | 189.6 |
| **19.7** | 164.7 | 166.1 | 168.4 | 172.2 | 176.6 | 181.2 | 185.4 | 188.0 | 189.7 |
| **19.8** | 164.7 | 166.2 | 168.4 | 172.3 | 176.7 | 181.2 | 185.5 | 188.0 | 189.8 |
| **19.9** | 164.8 | 166.2 | 168.5 | 172.3 | 176.7 | 181.3 | 185.5 | 188.1 | 189.8 |
| **20.0** | 164.8 | 166.3 | 168.5 | 172.3 | 176.7 | 181.3 | 185.6 | 188.2 | 189.9 |

**TABLE C. Women's weight**

| **Weight percentiles for women** | | | | | | | | | |
| --- | --- | --- | --- | --- | --- | --- | --- | --- | --- |
| **Age** | **P3** | **P5** | **P10** | **P25** | **P50** | **75** | **P90** | **P95** | **P97** |
| **3.0** | 11.1 | 11.4 | 11.8 | 12.7 | 13.7 | 15.0 | 16.3 | 17.2 | 17.8 |
| **3.1** | 11.2 | 11.5 | 12.0 | 12.9 | 14.0 | 15.3 | 16.7 | 17.6 | 18.2 |
| **3.2** | 11.4 | 11.7 | 12.2 | 13.1 | 14.3 | 15.6 | 17.0 | 18.0 | 18.7 |
| **3.3** | 11.6 | 11.9 | 12.4 | 13.3 | 14.5 | 15.9 | 17.4 | 18.4 | 19.1 |
| **3.4** | 11.8 | 12.1 | 12.6 | 13.6 | 14.8 | 16.2 | 17.8 | 18.8 | 19.5 |
| **3.5** | 12.0 | 12.3 | 12.8 | 13.8 | 15.1 | 16.5 | 18.1 | 19.2 | 20.0 |
| **3.6** | 12.1 | 12.5 | 13.0 | 14.0 | 15.3 | 16.9 | 18.5 | 19.7 | 20.5 |
| **3.7** | 12.3 | 12.7 | 13.2 | 14.2 | 15.6 | 17.2 | 18.9 | 20.1 | 20.9 |
| **3.8** | 12.5 | 12.8 | 13.4 | 14.5 | 15.9 | 17.5 | 19.3 | 20.5 | 21.4 |
| **3.9** | 12.7 | 13.0 | 13.6 | 14.7 | 16.1 | 17.8 | 19.7 | 21.0 | 21.9 |
| **4.0** | 12.9 | 13.2 | 13.8 | 14.9 | 16.4 | 18.1 | 20.1 | 21.4 | 22.4 |
| **4.1** | 13.0 | 13.4 | 14.0 | 15.2 | 16.7 | 18.5 | 20.5 | 21.9 | 22.9 |
| **4.2** | 13.2 | 13.6 | 14.2 | 15.4 | 16.9 | 18.8 | 20.9 | 22.3 | 23.4 |
| **4.3** | 13.4 | 13.8 | 14.4 | 15.6 | 17.2 | 19.1 | 21.3 | 22.8 | 23.9 |
| **4.4** | 13.6 | 14.0 | 14.6 | 15.8 | 17.5 | 19.5 | 21.7 | 23.2 | 24.4 |
| **4.5** | 13.7 | 14.1 | 14.8 | 16.1 | 17.7 | 19.8 | 22.1 | 23.7 | 24.9 |
| **4.6** | 13.9 | 14.3 | 15.0 | 16.3 | 18.0 | 20.1 | 22.5 | 24.2 | 25.4 |
| **4.7** | 14.1 | 14.5 | 15.2 | 16.5 | 18.3 | 20.5 | 22.9 | 24.7 | 26.0 |
| **4.8** | 14.3 | 14.7 | 15.4 | 16.8 | 18.6 | 20.8 | 23.3 | 25.2 | 26.5 |
| **4.9** | 14.4 | 14.9 | 15.6 | 17.0 | 18.8 | 21.1 | 23.8 | 25.7 | 27.1 |
| **5.0** | 14.6 | 15.1 | 15.8 | 17.2 | 19.1 | 21.5 | 24.2 | 26.2 | 27.6 |
| **5.1** | 14.8 | 15.2 | 16.0 | 17.5 | 19.4 | 21.8 | 24.6 | 26.7 | 28.2 |
| **5.2** | 15.0 | 15.4 | 16.2 | 17.7 | 19.7 | 22.2 | 25.1 | 27.2 | 28.7 |
| **5.3** | 15.1 | 15.6 | 16.4 | 17.9 | 20.0 | 22.6 | 25.5 | 27.7 | 29.3 |
| **5.4** | 15.3 | 15.8 | 16.6 | 18.2 | 20.3 | 22.9 | 26.0 | 28.2 | 29.9 |
| **5.5** | 15.5 | 16.0 | 16.8 | 18.4 | 20.6 | 23.3 | 26.4 | 28.7 | 30.5 |
| **5.6** | 15.7 | 16.2 | 17.0 | 18.6 | 20.8 | 23.6 | 26.9 | 29.3 | 31.1 |
| **5.7** | 15.9 | 16.4 | 17.2 | 18.9 | 21.1 | 24.0 | 27.3 | 29.8 | 31.7 |
| **5.8** | 16.0 | 16.6 | 17.4 | 19.1 | 21.4 | 24.4 | 27.8 | 30.4 | 32.3 |
| **5.9** | 16.2 | 16.8 | 17.6 | 19.4 | 21.7 | 24.8 | 28.3 | 30.9 | 32.9 |
| **6.0** | 16.4 | 16.9 | 17.9 | 19.6 | 22.1 | 25.1 | 28.8 | 31.5 | 33.5 |
| **6.1** | 16.6 | 17.1 | 18.1 | 19.9 | 22.4 | 25.5 | 29.3 | 32.0 | 34.2 |
| **6.2** | 16.8 | 17.3 | 18.3 | 20.1 | 22.7 | 25.9 | 29.7 | 32.6 | 34.8 |
| **6.3** | 16.9 | 17.5 | 18.5 | 20.4 | 23.0 | 26.3 | 30.2 | 33.2 | 35.4 |
| **6.4** | 17.1 | 17.7 | 18.7 | 20.6 | 23.3 | 26.7 | 30.7 | 33.8 | 36.1 |
| **6.5** | 17.3 | 17.9 | 18.9 | 20.9 | 23.6 | 27.1 | 31.2 | 34.4 | 36.7 |
| **6.6** | 17.5 | 18.1 | 19.2 | 21.2 | 23.9 | 27.5 | 31.8 | 34.9 | 37.4 |
| **6.7** | 17.7 | 18.3 | 19.4 | 21.4 | 24.3 | 27.9 | 32.3 | 35.5 | 38.0 |
| **6.8** | 17.9 | 18.5 | 19.6 | 21.7 | 24.6 | 28.4 | 32.8 | 36.1 | 38.7 |
| **6.9** | 18.1 | 18.7 | 19.8 | 22.0 | 24.9 | 28.8 | 33.3 | 36.7 | 39.4 |
| **7.0** | 18.3 | 18.9 | 20.1 | 22.3 | 25.3 | 29.2 | 33.8 | 37.4 | 40.0 |
| **7.1** | 18.5 | 19.2 | 20.3 | 22.5 | 25.6 | 29.6 | 34.4 | 38.0 | 40.7 |
| **7.2** | 18.7 | 19.4 | 20.5 | 22.8 | 26.0 | 30.1 | 34.9 | 38.6 | 41.4 |
| **7.3** | 18.9 | 19.6 | 20.8 | 23.1 | 26.3 | 30.5 | 35.5 | 39.2 | 42.0 |
| **7.4** | 19.1 | 19.8 | 21.0 | 23.4 | 26.7 | 30.9 | 36.0 | 39.8 | 42.7 |
| **7.5** | 19.3 | 20.0 | 21.3 | 23.7 | 27.0 | 31.4 | 36.5 | 40.4 | 43.4 |
| **7.6** | 19.5 | 20.2 | 21.5 | 24.0 | 27.4 | 31.8 | 37.1 | 41.1 | 44.1 |
| **7.7** | 19.7 | 20.4 | 21.7 | 24.3 | 27.8 | 32.3 | 37.7 | 41.7 | 44.7 |
| **7.8** | 19.9 | 20.7 | 22.0 | 24.6 | 28.1 | 32.8 | 38.2 | 42.3 | 45.4 |
| **7.9** | 20.1 | 20.9 | 22.2 | 24.9 | 28.5 | 33.2 | 38.8 | 43.0 | 46.1 |
| **8.0** | 20.3 | 21.1 | 22.5 | 25.2 | 28.9 | 33.7 | 39.4 | 43.6 | 46.8 |
| **8.1** | 20.5 | 21.4 | 22.8 | 25.5 | 29.3 | 34.2 | 39.9 | 44.2 | 47.5 |
| **8.2** | 20.7 | 21.6 | 23.0 | 25.8 | 29.7 | 34.7 | 40.5 | 44.9 | 48.2 |
| **8.3** | 21.0 | 21.8 | 23.3 | 26.1 | 30.1 | 35.1 | 41.1 | 45.5 | 48.9 |
| **8.4** | 21.2 | 22.1 | 23.6 | 26.5 | 30.5 | 35.6 | 41.7 | 46.2 | 49.6 |
| **8.5** | 21.4 | 22.3 | 23.8 | 26.8 | 30.9 | 36.1 | 42.3 | 46.8 | 50.3 |
| **8.6** | 21.6 | 22.5 | 24.1 | 27.1 | 31.3 | 36.6 | 42.9 | 47.5 | 51.0 |
| **8.7** | 21.9 | 22.8 | 24.4 | 27.4 | 31.7 | 37.1 | 43.5 | 48.2 | 51.7 |
| **8.8** | 22.1 | 23.0 | 24.7 | 27.8 | 32.1 | 37.6 | 44.1 | 48.8 | 52.4 |
| **8.9** | 22.3 | 23.3 | 24.9 | 28.1 | 32.5 | 38.1 | 44.7 | 49.5 | 53.1 |
| **9.0** | 22.6 | 23.6 | 25.2 | 28.5 | 32.9 | 38.6 | 45.3 | 50.2 | 53.8 |
| **9.1** | 22.8 | 23.8 | 25.5 | 28.8 | 33.4 | 39.2 | 45.9 | 50.8 | 54.5 |
| **9.2** | 23.1 | 24.1 | 25.8 | 29.2 | 33.8 | 39.7 | 46.5 | 51.5 | 55.2 |
| **9.3** | 23.3 | 24.4 | 26.1 | 29.5 | 34.2 | 40.2 | 47.1 | 52.2 | 55.9 |
| **9.4** | 23.6 | 24.6 | 26.4 | 29.9 | 34.6 | 40.7 | 47.7 | 52.8 | 56.6 |
| **9.5** | 23.8 | 24.9 | 26.7 | 30.2 | 35.1 | 41.2 | 48.3 | 53.5 | 57.3 |
| **9.6** | 24.1 | 25.2 | 27.0 | 30.6 | 35.5 | 41.8 | 48.9 | 54.2 | 58.1 |
| **9.7** | 24.4 | 25.5 | 27.3 | 31.0 | 36.0 | 42.3 | 49.5 | 54.8 | 58.8 |
| **9.8** | 24.6 | 25.7 | 27.7 | 31.3 | 36.4 | 42.8 | 50.2 | 55.5 | 59.5 |
| **9.9** | 24.9 | 26.0 | 28.0 | 31.7 | 36.8 | 43.3 | 50.8 | 56.2 | 60.2 |
| **10.0** | 25.2 | 26.3 | 28.3 | 32.1 | 37.3 | 43.9 | 51.4 | 56.8 | 60.9 |
| **10.1** | 25.4 | 26.6 | 28.6 | 32.5 | 37.7 | 44.4 | 52.0 | 57.5 | 61.6 |
| **10.2** | 25.7 | 26.9 | 28.9 | 32.8 | 38.2 | 44.9 | 52.6 | 58.2 | 62.3 |
| **10.3** | 26.0 | 27.2 | 29.3 | 33.2 | 38.6 | 45.4 | 53.2 | 58.8 | 63.0 |
| **10.4** | 26.3 | 27.5 | 29.6 | 33.6 | 39.1 | 46.0 | 53.8 | 59.5 | 63.7 |
| **10.5** | 26.6 | 27.8 | 29.9 | 34.0 | 39.5 | 46.5 | 54.4 | 60.1 | 64.4 |
| **10.6** | 26.9 | 28.2 | 30.3 | 34.4 | 40.0 | 47.0 | 55.0 | 60.8 | 65.0 |
| **10.7** | 27.2 | 28.5 | 30.6 | 34.8 | 40.4 | 47.5 | 55.6 | 61.4 | 65.7 |
| **10.8** | 27.5 | 28.8 | 31.0 | 35.2 | 40.9 | 48.0 | 56.2 | 62.1 | 66.4 |
| **10.9** | 27.8 | 29.1 | 31.3 | 35.5 | 41.3 | 48.6 | 56.8 | 62.7 | 67.1 |
| **11.0** | 28.1 | 29.4 | 31.6 | 35.9 | 41.8 | 49.1 | 57.4 | 63.3 | 67.7 |
| **11.1** | 28.4 | 29.8 | 32.0 | 36.3 | 42.2 | 49.6 | 57.9 | 64.0 | 68.4 |
| **11.2** | 28.7 | 30.1 | 32.3 | 36.7 | 42.6 | 50.1 | 58.5 | 64.6 | 69.0 |
| **11.3** | 29.0 | 30.4 | 32.7 | 37.1 | 43.1 | 50.6 | 59.1 | 65.2 | 69.7 |
| **11.4** | 29.4 | 30.7 | 33.0 | 37.5 | 43.5 | 51.1 | 59.6 | 65.8 | 70.3 |
| **11.5** | 29.7 | 31.1 | 33.4 | 37.9 | 43.9 | 51.6 | 60.2 | 66.4 | 70.9 |
| **11.6** | 30.0 | 31.4 | 33.7 | 38.2 | 44.4 | 52.0 | 60.7 | 66.9 | 71.5 |
| **11.7** | 30.3 | 31.7 | 34.1 | 38.6 | 44.8 | 52.5 | 61.2 | 67.5 | 72.1 |
| **11.8** | 30.6 | 32.1 | 34.4 | 39.0 | 45.2 | 53.0 | 61.8 | 68.1 | 72.7 |
| **11.9** | 31.0 | 32.4 | 34.8 | 39.4 | 45.6 | 53.4 | 62.3 | 68.6 | 73.2 |
| **12.0** | 31.3 | 32.7 | 35.1 | 39.8 | 46.0 | 53.9 | 62.8 | 69.1 | 73.8 |
| **12.1** | 31.6 | 33.0 | 35.5 | 40.1 | 46.4 | 54.3 | 63.2 | 69.6 | 74.3 |
| **12.2** | 31.9 | 33.4 | 35.8 | 40.5 | 46.8 | 54.8 | 63.7 | 70.1 | 74.8 |
| **12.3** | 32.2 | 33.7 | 36.1 | 40.9 | 47.2 | 55.2 | 64.2 | 70.6 | 75.3 |
| **12.4** | 32.6 | 34.0 | 36.5 | 41.2 | 47.6 | 55.6 | 64.6 | 71.1 | 75.8 |
| **12.5** | 32.9 | 34.4 | 36.8 | 41.6 | 48.0 | 56.0 | 65.1 | 71.5 | 76.3 |
| **12.6** | 33.2 | 34.7 | 37.2 | 41.9 | 48.4 | 56.4 | 65.5 | 72.0 | 76.7 |
| **12.7** | 33.5 | 35.0 | 37.5 | 42.3 | 48.7 | 56.8 | 65.9 | 72.4 | 77.1 |
| **12.8** | 33.8 | 35.3 | 37.8 | 42.6 | 49.1 | 57.2 | 66.3 | 72.8 | 77.6 |
| **12.9** | 34.2 | 35.6 | 38.1 | 43.0 | 49.5 | 57.5 | 66.7 | 73.2 | 78.0 |
| **13.0** | 34.5 | 36.0 | 38.5 | 43.3 | 49.8 | 57.9 | 67.0 | 73.6 | 78.3 |
| **13.1** | 34.8 | 36.3 | 38.8 | 43.6 | 50.1 | 58.2 | 67.4 | 73.9 | 78.7 |
| **13.2** | 35.1 | 36.6 | 39.1 | 43.9 | 50.5 | 58.6 | 67.7 | 74.3 | 79.1 |
| **13.3** | 35.4 | 36.9 | 39.4 | 44.3 | 50.8 | 58.9 | 68.0 | 74.6 | 79.4 |
| **13.4** | 35.7 | 37.2 | 39.7 | 44.6 | 51.1 | 59.2 | 68.3 | 74.9 | 79.7 |
| **13.5** | 36.0 | 37.5 | 40.0 | 44.9 | 51.4 | 59.5 | 68.6 | 75.2 | 80.0 |
| **13.6** | 36.3 | 37.8 | 40.3 | 45.2 | 51.7 | 59.8 | 68.9 | 75.5 | 80.3 |
| **13.7** | 36.6 | 38.1 | 40.6 | 45.5 | 52.0 | 60.0 | 69.2 | 75.7 | 80.5 |
| **13.8** | 36.9 | 38.4 | 40.9 | 45.7 | 52.2 | 60.3 | 69.4 | 76.0 | 80.7 |
| **13.9** | 37.2 | 38.7 | 41.2 | 46.0 | 52.5 | 60.6 | 69.6 | 76.2 | 81.0 |
| **14.0** | 37.5 | 39.0 | 41.5 | 46.3 | 52.8 | 60.8 | 69.9 | 76.4 | 81.2 |
| **14.1** | 37.8 | 39.3 | 41.8 | 46.6 | 53.0 | 61.0 | 70.1 | 76.6 | 81.4 |
| **14.2** | 38.1 | 39.6 | 42.1 | 46.8 | 53.2 | 61.2 | 70.3 | 76.8 | 81.5 |
| **14.3** | 38.4 | 39.8 | 42.3 | 47.1 | 53.5 | 61.4 | 70.4 | 76.9 | 81.7 |
| **14.4** | 38.6 | 40.1 | 42.6 | 47.3 | 53.7 | 61.6 | 70.6 | 77.1 | 81.8 |
| **14.5** | 38.9 | 40.4 | 42.8 | 47.6 | 53.9 | 61.8 | 70.8 | 77.2 | 81.9 |
| **14.6** | 39.2 | 40.6 | 43.1 | 47.8 | 54.1 | 62.0 | 70.9 | 77.3 | 82.1 |
| **14.7** | 39.4 | 40.9 | 43.3 | 48.0 | 54.3 | 62.2 | 71.0 | 77.4 | 82.1 |
| **14.8** | 39.7 | 41.1 | 43.6 | 48.2 | 54.5 | 62.3 | 71.2 | 77.5 | 82.2 |
| **14.9** | 39.9 | 41.4 | 43.8 | 48.4 | 54.7 | 62.5 | 71.3 | 77.6 | 82.3 |
| **15.0** | 40.2 | 41.6 | 44.0 | 48.6 | 54.9 | 62.6 | 71.4 | 77.7 | 82.3 |
| **15.1** | 40.4 | 41.9 | 44.3 | 48.8 | 55.0 | 62.7 | 71.4 | 77.7 | 82.4 |
| **15.2** | 40.6 | 42.1 | 44.5 | 49.0 | 55.2 | 62.8 | 71.5 | 77.8 | 82.4 |
| **15.3** | 40.9 | 42.3 | 44.7 | 49.2 | 55.3 | 63.0 | 71.6 | 77.8 | 82.4 |
| **15.4** | 41.1 | 42.5 | 44.9 | 49.4 | 55.5 | 63.1 | 71.6 | 77.8 | 82.4 |
| **15.5** | 41.3 | 42.7 | 45.1 | 49.6 | 55.6 | 63.1 | 71.7 | 77.8 | 82.4 |
| **15.6** | 41.5 | 42.9 | 45.3 | 49.7 | 55.7 | 63.2 | 71.7 | 77.8 | 82.4 |
| **15.7** | 41.7 | 43.1 | 45.5 | 49.9 | 55.9 | 63.3 | 71.7 | 77.8 | 82.3 |
| **15.8** | 41.9 | 43.3 | 45.6 | 50.0 | 56.0 | 63.4 | 71.7 | 77.8 | 82.3 |
| **15.9** | 42.1 | 43.5 | 45.8 | 50.2 | 56.1 | 63.4 | 71.8 | 77.8 | 82.2 |
| **16.0** | 42.3 | 43.7 | 46.0 | 50.3 | 56.2 | 63.5 | 71.8 | 77.7 | 82.1 |
| **16.1** | 42.5 | 43.9 | 46.1 | 50.5 | 56.3 | 63.5 | 71.7 | 77.7 | 82.1 |
| **16.2** | 42.7 | 44.0 | 46.3 | 50.6 | 56.4 | 63.6 | 71.7 | 77.6 | 82.0 |
| **16.3** | 42.8 | 44.2 | 46.4 | 50.7 | 56.5 | 63.6 | 71.7 | 77.6 | 81.9 |
| **16.4** | 43.0 | 44.3 | 46.6 | 50.8 | 56.6 | 63.7 | 71.7 | 77.5 | 81.8 |
| **16.5** | 43.2 | 44.5 | 46.7 | 51.0 | 56.6 | 63.7 | 71.7 | 77.4 | 81.7 |
| **16.6** | 43.3 | 44.6 | 46.8 | 51.1 | 56.7 | 63.7 | 71.6 | 77.4 | 81.6 |
| **16.7** | 43.4 | 44.8 | 47.0 | 51.2 | 56.8 | 63.7 | 71.6 | 77.3 | 81.5 |
| **16.8** | 43.6 | 44.9 | 47.1 | 51.3 | 56.8 | 63.8 | 71.5 | 77.2 | 81.3 |
| **16.9** | 43.7 | 45.0 | 47.2 | 51.4 | 56.9 | 63.8 | 71.5 | 77.1 | 81.2 |
| **17.0** | 43.8 | 45.2 | 47.3 | 51.4 | 57.0 | 63.8 | 71.5 | 77.0 | 81.1 |
| **17.1** | 44.0 | 45.3 | 47.4 | 51.5 | 57.0 | 63.8 | 71.4 | 76.9 | 80.9 |
| **17.2** | 44.1 | 45.4 | 47.5 | 51.6 | 57.1 | 63.8 | 71.4 | 76.8 | 80.8 |
| **17.3** | 44.2 | 45.5 | 47.6 | 51.7 | 57.1 | 63.8 | 71.3 | 76.7 | 80.7 |
| **17.4** | 44.3 | 45.6 | 47.7 | 51.8 | 57.1 | 63.8 | 71.2 | 76.6 | 80.5 |
| **17.5** | 44.4 | 45.7 | 47.8 | 51.8 | 57.2 | 63.8 | 71.2 | 76.5 | 80.4 |
| **17.6** | 44.5 | 45.8 | 47.9 | 51.9 | 57.2 | 63.8 | 71.1 | 76.4 | 80.3 |
| **17.7** | 44.6 | 45.9 | 48.0 | 52.0 | 57.3 | 63.8 | 71.1 | 76.3 | 80.1 |
| **17.8** | 44.7 | 46.0 | 48.0 | 52.0 | 57.3 | 63.8 | 71.0 | 76.2 | 80.0 |
| **17.9** | 44.8 | 46.0 | 48.1 | 52.1 | 57.3 | 63.8 | 70.9 | 76.1 | 79.9 |
| **18.0** | 44.9 | 46.1 | 48.2 | 52.1 | 57.3 | 63.7 | 70.9 | 76.0 | 79.7 |
| **18.1** | 44.9 | 46.2 | 48.3 | 52.2 | 57.4 | 63.7 | 70.8 | 75.9 | 79.6 |
| **18.2** | 45.0 | 46.3 | 48.3 | 52.2 | 57.4 | 63.7 | 70.8 | 75.8 | 79.5 |
| **18.3** | 45.1 | 46.3 | 48.4 | 52.3 | 57.4 | 63.7 | 70.7 | 75.7 | 79.3 |
| **18.4** | 45.1 | 46.4 | 48.4 | 52.3 | 57.4 | 63.7 | 70.6 | 75.6 | 79.2 |
| **18.5** | 45.2 | 46.4 | 48.5 | 52.4 | 57.5 | 63.7 | 70.6 | 75.5 | 79.1 |
| **18.6** | 45.3 | 46.5 | 48.5 | 52.4 | 57.5 | 63.7 | 70.5 | 75.4 | 79.0 |
| **18.7** | 45.3 | 46.6 | 48.6 | 52.4 | 57.5 | 63.6 | 70.5 | 75.3 | 78.8 |
| **18.8** | 45.4 | 46.6 | 48.6 | 52.5 | 57.5 | 63.6 | 70.4 | 75.2 | 78.7 |
| **18.9** | 45.4 | 46.7 | 48.7 | 52.5 | 57.5 | 63.6 | 70.4 | 75.1 | 78.6 |
| **19.0** | 45.5 | 46.7 | 48.7 | 52.5 | 57.5 | 63.6 | 70.3 | 75.1 | 78.5 |
| **19.1** | 45.5 | 46.7 | 48.8 | 52.5 | 57.5 | 63.6 | 70.2 | 75.0 | 78.4 |
| **19.2** | 45.6 | 46.8 | 48.8 | 52.6 | 57.5 | 63.6 | 70.2 | 74.9 | 78.3 |
| **19.3** | 45.6 | 46.8 | 48.8 | 52.6 | 57.5 | 63.5 | 70.1 | 74.8 | 78.2 |
| **19.4** | 45.7 | 46.9 | 48.9 | 52.6 | 57.5 | 63.5 | 70.1 | 74.7 | 78.1 |
| **19.5** | 45.7 | 46.9 | 48.9 | 52.6 | 57.5 | 63.5 | 70.0 | 74.7 | 78.0 |
| **19.6** | 45.7 | 46.9 | 48.9 | 52.7 | 57.5 | 63.5 | 70.0 | 74.6 | 77.9 |
| **19.7** | 45.8 | 47.0 | 48.9 | 52.7 | 57.6 | 63.5 | 70.0 | 74.5 | 77.8 |
| **19.8** | 45.8 | 47.0 | 49.0 | 52.7 | 57.6 | 63.4 | 69.9 | 74.5 | 77.8 |
| **19.9** | 45.8 | 47.0 | 49.0 | 52.7 | 57.5 | 63.4 | 69.9 | 74.4 | 77.7 |
| **20.0** | 45.8 | 47.0 | 49.0 | 52.7 | 57.5 | 63.4 | 69.8 | 74.3 | 77.6 |

**TABLE D. Men's weight**

| **Weight percentiles for men** | | | | | | | | | |
| --- | --- | --- | --- | --- | --- | --- | --- | --- | --- |
| **Age** | **P3** | **P5** | **P10** | **P25** | **P50** | **P75** | **P90** | **P95** | **P97** |
| **3.0** | 11.5 | 11.8 | 12.3 | 13.3 | 14.4 | 15.6 | 16.8 | 17.6 | 18.1 |
| **3.1** | 11.7 | 12.0 | 12.5 | 13.5 | 14.6 | 15.9 | 17.1 | 18.0 | 18.5 |
| **3.2** | 11.9 | 12.2 | 12.7 | 13.7 | 14.9 | 16.2 | 17.5 | 18.3 | 18.9 |
| **3.3** | 12.0 | 12.4 | 12.9 | 13.9 | 15.1 | 16.5 | 17.9 | 18.7 | 19.3 |
| **3.4** | 12.2 | 12.6 | 13.1 | 14.1 | 15.4 | 16.8 | 18.2 | 19.1 | 19.8 |
| **3.5** | 12.4 | 12.7 | 13.3 | 14.4 | 15.6 | 17.1 | 18.6 | 19.5 | 20.2 |
| **3.6** | 12.6 | 12.9 | 13.5 | 14.6 | 15.9 | 17.4 | 18.9 | 19.9 | 20.6 |
| **3.7** | 12.7 | 13.1 | 13.7 | 14.8 | 16.2 | 17.7 | 19.3 | 20.3 | 21.1 |
| **3.8** | 12.9 | 13.3 | 13.9 | 15.0 | 16.4 | 18.0 | 19.7 | 20.8 | 21.5 |
| **3.9** | 13.1 | 13.5 | 14.1 | 15.2 | 16.7 | 18.3 | 20.0 | 21.2 | 22.0 |
| **4.0** | 13.3 | 13.6 | 14.3 | 15.4 | 16.9 | 18.6 | 20.4 | 21.6 | 22.5 |
| **4.1** | 13.4 | 13.8 | 14.5 | 15.7 | 17.2 | 19.0 | 20.8 | 22.1 | 22.9 |
| **4.2** | 13.6 | 14.0 | 14.7 | 15.9 | 17.4 | 19.3 | 21.2 | 22.5 | 23.4 |
| **4.3** | 13.8 | 14.2 | 14.9 | 16.1 | 17.7 | 19.6 | 21.6 | 22.9 | 23.9 |
| **4.4** | 14.0 | 14.4 | 15.1 | 16.3 | 18.0 | 19.9 | 22.0 | 23.4 | 24.4 |
| **4.5** | 14.1 | 14.6 | 15.3 | 16.6 | 18.2 | 20.2 | 22.4 | 23.9 | 24.9 |
| **4.6** | 14.3 | 14.7 | 15.5 | 16.8 | 18.5 | 20.6 | 22.8 | 24.3 | 25.4 |
| **4.7** | 14.5 | 14.9 | 15.7 | 17.0 | 18.8 | 20.9 | 23.2 | 24.8 | 25.9 |
| **4.8** | 14.7 | 15.1 | 15.9 | 17.2 | 19.0 | 21.2 | 23.6 | 25.3 | 26.5 |
| **4.9** | 14.9 | 15.3 | 16.0 | 17.5 | 19.3 | 21.6 | 24.0 | 25.8 | 27.0 |
| **5.0** | 15.0 | 15.5 | 16.2 | 17.7 | 19.6 | 21.9 | 24.5 | 26.3 | 27.6 |
| **5.1** | 15.2 | 15.7 | 16.5 | 17.9 | 19.9 | 22.2 | 24.9 | 26.8 | 28.1 |
| **5.2** | 15.4 | 15.9 | 16.7 | 18.1 | 20.1 | 22.6 | 25.3 | 27.3 | 28.7 |
| **5.3** | 15.6 | 16.1 | 16.9 | 18.4 | 20.4 | 22.9 | 25.8 | 27.8 | 29.3 |
| **5.4** | 15.8 | 16.2 | 17.1 | 18.6 | 20.7 | 23.3 | 26.2 | 28.3 | 29.9 |
| **5.5** | 15.9 | 16.4 | 17.3 | 18.8 | 21.0 | 23.6 | 26.7 | 28.9 | 30.5 |
| **5.6** | 16.1 | 16.6 | 17.5 | 19.1 | 21.3 | 24.0 | 27.1 | 29.4 | 31.1 |
| **5.7** | 16.3 | 16.8 | 17.7 | 19.3 | 21.5 | 24.4 | 27.6 | 30.0 | 31.8 |
| **5.8** | 16.5 | 17.0 | 17.9 | 19.6 | 21.8 | 24.7 | 28.1 | 30.5 | 32.4 |
| **5.9** | 16.7 | 17.2 | 18.1 | 19.8 | 22.1 | 25.1 | 28.5 | 31.1 | 33.1 |
| **6.0** | 16.9 | 17.4 | 18.3 | 20.0 | 22.4 | 25.5 | 29.0 | 31.7 | 33.7 |
| **6.1** | 17.1 | 17.6 | 18.5 | 20.3 | 22.7 | 25.8 | 29.5 | 32.3 | 34.4 |
| **6.2** | 17.2 | 17.8 | 18.7 | 20.5 | 23.0 | 26.2 | 30.0 | 32.9 | 35.1 |
| **6.3** | 17.4 | 18.0 | 18.9 | 20.8 | 23.3 | 26.6 | 30.5 | 33.5 | 35.8 |
| **6.4** | 17.6 | 18.2 | 19.2 | 21.0 | 23.6 | 27.0 | 31.0 | 34.1 | 36.5 |
| **6.5** | 17.8 | 18.4 | 19.4 | 21.3 | 23.9 | 27.4 | 31.5 | 34.7 | 37.2 |
| **6.6** | 18.0 | 18.6 | 19.6 | 21.5 | 24.2 | 27.8 | 32.1 | 35.4 | 37.9 |
| **6.7** | 18.2 | 18.8 | 19.8 | 21.8 | 24.6 | 28.2 | 32.6 | 36.0 | 38.6 |
| **6.8** | 18.4 | 19.0 | 20.0 | 22.0 | 24.9 | 28.6 | 33.1 | 36.6 | 39.4 |
| **6.9** | 18.6 | 19.2 | 20.2 | 22.3 | 25.2 | 29.0 | 33.6 | 37.3 | 40.1 |
| **7.0** | 18.8 | 19.4 | 20.5 | 22.6 | 25.5 | 29.4 | 34.2 | 37.9 | 40.9 |
| **7.1** | 19.0 | 19.6 | 20.7 | 22.8 | 25.8 | 29.8 | 34.7 | 38.6 | 41.6 |
| **7.2** | 19.2 | 19.8 | 20.9 | 23.1 | 26.2 | 30.2 | 35.3 | 39.3 | 42.4 |
| **7.3** | 19.4 | 20.0 | 21.1 | 23.4 | 26.5 | 30.7 | 35.8 | 39.9 | 43.1 |
| **7.4** | 19.5 | 20.2 | 21.4 | 23.6 | 26.8 | 31.1 | 36.4 | 40.6 | 43.9 |
| **7.5** | 19.7 | 20.4 | 21.6 | 23.9 | 27.2 | 31.5 | 37.0 | 41.3 | 44.7 |
| **7.6** | 19.9 | 20.6 | 21.8 | 24.2 | 27.5 | 32.0 | 37.5 | 42.0 | 45.5 |
| **7.7** | 20.1 | 20.9 | 22.1 | 24.5 | 27.9 | 32.4 | 38.1 | 42.6 | 46.2 |
| **7.8** | 20.3 | 21.1 | 22.3 | 24.7 | 28.2 | 32.9 | 38.7 | 43.3 | 47.0 |
| **7.9** | 20.5 | 21.3 | 22.5 | 25.0 | 28.6 | 33.3 | 39.3 | 44.0 | 47.8 |
| **8.0** | 20.7 | 21.5 | 22.8 | 25.3 | 28.9 | 33.8 | 39.8 | 44.7 | 48.6 |
| **8.1** | 21.0 | 21.7 | 23.0 | 25.6 | 29.3 | 34.2 | 40.4 | 45.4 | 49.4 |
| **8.2** | 21.2 | 21.9 | 23.3 | 25.9 | 29.6 | 34.7 | 41.0 | 46.1 | 50.1 |
| **8.3** | 21.4 | 22.2 | 23.5 | 26.2 | 30.0 | 35.1 | 41.6 | 46.8 | 50.9 |
| **8.4** | 21.6 | 22.4 | 23.8 | 26.5 | 30.4 | 35.6 | 42.2 | 47.5 | 51.7 |
| **8.5** | 21.8 | 22.6 | 24.0 | 26.8 | 30.7 | 36.1 | 42.8 | 48.2 | 52.5 |
| **8.6** | 22.0 | 22.8 | 24.2 | 27.1 | 31.1 | 36.6 | 43.4 | 48.9 | 53.2 |
| **8.7** | 22.2 | 23.0 | 24.5 | 27.4 | 31.5 | 37.0 | 44.0 | 49.6 | 54.0 |
| **8.8** | 22.4 | 23.3 | 24.8 | 27.7 | 31.9 | 37.5 | 44.6 | 50.3 | 54.7 |
| **8.9** | 22.6 | 23.5 | 25.0 | 28.0 | 32.3 | 38.0 | 45.2 | 51.0 | 55.5 |
| **9.0** | 22.8 | 23.7 | 25.3 | 28.3 | 32.7 | 38.5 | 45.8 | 51.6 | 56.3 |
| **9.1** | 23.0 | 24.0 | 25.5 | 28.6 | 33.1 | 39.0 | 46.4 | 52.3 | 57.0 |
| **9.2** | 23.3 | 24.2 | 25.8 | 29.0 | 33.4 | 39.5 | 47.0 | 53.0 | 57.7 |
| **9.3** | 23.5 | 24.4 | 26.1 | 29.3 | 33.8 | 40.0 | 47.7 | 53.7 | 58.5 |
| **9.4** | 23.7 | 24.7 | 26.3 | 29.6 | 34.3 | 40.5 | 48.3 | 54.4 | 59.2 |
| **9.5** | 23.9 | 24.9 | 26.6 | 29.9 | 34.7 | 41.0 | 48.9 | 55.1 | 59.9 |
| **9.6** | 24.2 | 25.2 | 26.9 | 30.3 | 35.1 | 41.5 | 49.5 | 55.8 | 60.7 |
| **9.7** | 24.4 | 25.4 | 27.1 | 30.6 | 35.5 | 42.0 | 50.1 | 56.4 | 61.4 |
| **9.8** | 24.6 | 25.7 | 27.4 | 30.9 | 35.9 | 42.6 | 50.7 | 57.1 | 62.1 |
| **9.9** | 24.9 | 25.9 | 27.7 | 31.3 | 36.3 | 43.1 | 51.3 | 57.8 | 62.8 |
| **10.0** | 25.1 | 26.2 | 28.0 | 31.6 | 36.8 | 43.6 | 52.0 | 58.5 | 63.5 |
| **10.1** | 25.3 | 26.4 | 28.3 | 32.0 | 37.2 | 44.1 | 52.6 | 59.1 | 64.2 |
| **10.2** | 25.6 | 26.7 | 28.6 | 32.3 | 37.6 | 44.6 | 53.2 | 59.8 | 64.9 |
| **10.3** | 25.8 | 27.0 | 28.9 | 32.7 | 38.1 | 45.2 | 53.8 | 60.5 | 65.6 |
| **10.4** | 26.1 | 27.2 | 29.2 | 33.1 | 38.5 | 45.7 | 54.4 | 61.1 | 66.3 |
| **10.5** | 26.3 | 27.5 | 29.5 | 33.4 | 39.0 | 46.2 | 55.1 | 61.8 | 67.0 |
| **10.6** | 26.6 | 27.8 | 29.8 | 33.8 | 39.4 | 46.8 | 55.7 | 62.4 | 67.6 |
| **10.7** | 26.9 | 28.1 | 30.1 | 34.2 | 39.9 | 47.3 | 56.3 | 63.1 | 68.3 |
| **10.8** | 27.1 | 28.4 | 30.5 | 34.6 | 40.3 | 47.9 | 56.9 | 63.8 | 69.0 |
| **10.9** | 27.4 | 28.7 | 30.8 | 35.0 | 40.8 | 48.4 | 57.5 | 64.4 | 69.6 |
| **11.0** | 27.7 | 29.0 | 31.1 | 35.3 | 41.3 | 49.0 | 58.1 | 65.1 | 70.3 |
| **11.1** | 28.0 | 29.3 | 31.5 | 35.7 | 41.7 | 49.5 | 58.8 | 65.7 | 71.0 |
| **11.2** | 28.3 | 29.6 | 31.8 | 36.1 | 42.2 | 50.1 | 59.4 | 66.4 | 71.6 |
| **11.3** | 28.6 | 29.9 | 32.1 | 36.6 | 42.7 | 50.6 | 60.0 | 67.0 | 72.3 |
| **11.4** | 28.9 | 30.2 | 32.5 | 37.0 | 43.2 | 51.2 | 60.6 | 67.6 | 72.9 |
| **11.5** | 29.2 | 30.6 | 32.9 | 37.4 | 43.7 | 51.7 | 61.2 | 68.3 | 73.6 |
| **11.6** | 29.5 | 30.9 | 33.2 | 37.8 | 44.1 | 52.3 | 61.8 | 68.9 | 74.2 |
| **11.7** | 29.8 | 31.2 | 33.6 | 38.2 | 44.6 | 52.9 | 62.5 | 69.6 | 74.9 |
| **11.8** | 30.1 | 31.6 | 34.0 | 38.7 | 45.1 | 53.4 | 63.1 | 70.2 | 75.5 |
| **11.9** | 30.5 | 31.9 | 34.3 | 39.1 | 45.6 | 54.0 | 63.7 | 70.8 | 76.1 |
| **12.0** | 30.8 | 32.3 | 34.7 | 39.5 | 46.1 | 54.5 | 64.3 | 71.5 | 76.8 |
| **12.1** | 31.2 | 32.6 | 35.1 | 40.0 | 46.6 | 55.1 | 64.9 | 72.1 | 77.4 |
| **12.2** | 31.5 | 33.0 | 35.5 | 40.4 | 47.1 | 55.7 | 65.5 | 72.7 | 78.1 |
| **12.3** | 31.9 | 33.4 | 35.9 | 40.9 | 47.6 | 56.2 | 66.1 | 73.4 | 78.7 |
| **12.4** | 32.2 | 33.7 | 36.3 | 41.3 | 48.1 | 56.8 | 66.7 | 74.0 | 79.3 |
| **12.5** | 32.6 | 34.1 | 36.7 | 41.8 | 48.6 | 57.4 | 67.3 | 74.6 | 80.0 |
| **12.6** | 33.0 | 34.5 | 37.1 | 42.2 | 49.2 | 57.9 | 67.9 | 75.2 | 80.6 |
| **12.7** | 33.3 | 34.9 | 37.6 | 42.7 | 49.7 | 58.5 | 68.6 | 75.8 | 81.2 |
| **12.8** | 33.7 | 35.3 | 38.0 | 43.1 | 50.2 | 59.0 | 69.1 | 76.5 | 81.8 |
| **12.9** | 34.1 | 35.7 | 38.4 | 43.6 | 50.7 | 59.6 | 69.7 | 77.1 | 82.5 |
| **13.0** | 34.5 | 36.1 | 38.8 | 44.1 | 51.2 | 60.2 | 70.3 | 77.7 | 83.1 |
| **13.1** | 34.9 | 36.5 | 39.3 | 44.5 | 51.7 | 60.7 | 70.9 | 78.3 | 83.7 |
| **13.2** | 35.3 | 37.0 | 39.7 | 45.0 | 52.2 | 61.3 | 71.5 | 78.9 | 84.3 |
| **13.3** | 35.7 | 37.4 | 40.1 | 45.5 | 52.7 | 61.8 | 72.1 | 79.5 | 84.9 |
| **13.4** | 36.2 | 37.8 | 40.6 | 46.0 | 53.2 | 62.4 | 72.7 | 80.1 | 85.5 |
| **13.5** | 36.6 | 38.2 | 41.0 | 46.4 | 53.7 | 62.9 | 73.2 | 80.7 | 86.1 |
| **13.6** | 37.0 | 38.7 | 41.5 | 46.9 | 54.2 | 63.4 | 73.8 | 81.3 | 86.7 |
| **13.7** | 37.4 | 39.1 | 41.9 | 47.4 | 54.7 | 64.0 | 74.4 | 81.8 | 87.3 |
| **13.8** | 37.9 | 39.6 | 42.4 | 47.9 | 55.2 | 64.5 | 74.9 | 82.4 | 87.9 |
| **13.9** | 38.3 | 40.0 | 42.8 | 48.3 | 55.7 | 65.0 | 75.5 | 83.0 | 88.5 |
| **14.0** | 38.7 | 40.4 | 43.3 | 48.8 | 56.2 | 65.5 | 76.0 | 83.5 | 89.0 |
| **14.1** | 39.2 | 40.9 | 43.8 | 49.3 | 56.7 | 66.0 | 76.5 | 84.1 | 89.6 |
| **14.2** | 39.6 | 41.3 | 44.2 | 49.7 | 57.2 | 66.5 | 77.0 | 84.6 | 90.1 |
| **14.3** | 40.1 | 41.8 | 44.7 | 50.2 | 57.7 | 67.0 | 77.6 | 85.1 | 90.7 |
| **14.4** | 40.5 | 42.2 | 45.1 | 50.7 | 58.1 | 67.5 | 78.0 | 85.6 | 91.2 |
| **14.5** | 40.9 | 42.7 | 45.6 | 51.1 | 58.6 | 68.0 | 78.5 | 86.1 | 91.7 |
| **14.6** | 41.4 | 43.1 | 46.0 | 51.6 | 59.1 | 68.4 | 79.0 | 86.6 | 92.2 |
| **14.7** | 41.8 | 43.6 | 46.4 | 52.0 | 59.5 | 68.9 | 79.5 | 87.1 | 92.7 |
| **14.8** | 42.3 | 44.0 | 46.9 | 52.5 | 60.0 | 69.3 | 79.9 | 87.6 | 93.2 |
| **14.9** | 42.7 | 44.4 | 47.3 | 52.9 | 60.4 | 69.8 | 80.4 | 88.0 | 93.6 |
| **15.0** | 43.1 | 44.9 | 47.8 | 53.3 | 60.8 | 70.2 | 80.8 | 88.4 | 94.0 |
| **15.1** | 43.5 | 45.3 | 48.2 | 53.7 | 61.3 | 70.6 | 81.2 | 88.9 | 94.5 |
| **15.2** | 44.0 | 45.7 | 48.6 | 54.2 | 61.7 | 71.0 | 81.6 | 89.3 | 94.9 |
| **15.3** | 44.4 | 46.1 | 49.0 | 54.6 | 62.1 | 71.4 | 82.0 | 89.7 | 95.3 |
| **15.4** | 44.8 | 46.5 | 49.4 | 55.0 | 62.5 | 71.8 | 82.4 | 90.0 | 95.6 |
| **15.5** | 45.2 | 46.9 | 49.8 | 55.4 | 62.9 | 72.2 | 82.8 | 90.4 | 96.0 |
| **15.6** | 45.6 | 47.3 | 50.2 | 55.8 | 63.2 | 72.6 | 83.1 | 90.7 | 96.3 |
| **15.7** | 46.0 | 47.7 | 50.6 | 56.1 | 63.6 | 72.9 | 83.5 | 91.1 | 96.6 |
| **15.8** | 46.4 | 48.1 | 51.0 | 56.5 | 64.0 | 73.3 | 83.8 | 91.4 | 96.9 |
| **15.9** | 46.7 | 48.5 | 51.3 | 56.9 | 64.3 | 73.6 | 84.1 | 91.7 | 97.2 |
| **16.0** | 47.1 | 48.8 | 51.7 | 57.2 | 64.7 | 73.9 | 84.4 | 91.9 | 97.5 |
| **16.1** | 47.5 | 49.2 | 52.1 | 57.6 | 65.0 | 74.2 | 84.7 | 92.2 | 97.7 |
| **16.2** | 47.8 | 49.5 | 52.4 | 57.9 | 65.3 | 74.5 | 84.9 | 92.5 | 98.0 |
| **16.3** | 48.2 | 49.9 | 52.8 | 58.3 | 65.6 | 74.8 | 85.2 | 92.7 | 98.2 |
| **16.4** | 48.5 | 50.2 | 53.1 | 58.6 | 66.0 | 75.1 | 85.5 | 92.9 | 98.4 |
| **16.5** | 48.8 | 50.5 | 53.4 | 58.9 | 66.3 | 75.4 | 85.7 | 93.1 | 98.5 |
| **16.6** | 49.1 | 50.9 | 53.7 | 59.2 | 66.6 | 75.7 | 85.9 | 93.3 | 98.7 |
| **16.7** | 49.5 | 51.2 | 54.0 | 59.5 | 66.8 | 75.9 | 86.1 | 93.5 | 98.8 |
| **16.8** | 49.8 | 51.5 | 54.3 | 59.8 | 67.1 | 76.2 | 86.3 | 93.6 | 99.0 |
| **16.9** | 50.1 | 51.8 | 54.6 | 60.1 | 67.4 | 76.4 | 86.5 | 93.8 | 99.1 |
| **17.0** | 50.3 | 52.1 | 54.9 | 60.4 | 67.6 | 76.6 | 86.7 | 93.9 | 99.2 |
| **17.1** | 50.6 | 52.4 | 55.2 | 60.6 | 67.9 | 76.9 | 86.9 | 94.0 | 99.3 |
| **17.2** | 50.9 | 52.6 | 55.5 | 60.9 | 68.2 | 77.1 | 87.0 | 94.1 | 99.3 |
| **17.3** | 51.2 | 52.9 | 55.7 | 61.2 | 68.4 | 77.3 | 87.2 | 94.2 | 99.4 |
| **17.4** | 51.4 | 53.2 | 56.0 | 61.4 | 68.6 | 77.5 | 87.3 | 94.3 | 99.4 |
| **17.5** | 51.7 | 53.4 | 56.3 | 61.7 | 68.9 | 77.7 | 87.5 | 94.4 | 99.5 |
| **17.6** | 51.9 | 53.7 | 56.5 | 61.9 | 69.1 | 77.8 | 87.6 | 94.5 | 99.5 |
| **17.7** | 52.2 | 53.9 | 56.7 | 62.1 | 69.3 | 78.0 | 87.7 | 94.5 | 99.5 |
| **17.8** | 52.4 | 54.1 | 57.0 | 62.4 | 69.5 | 78.2 | 87.8 | 94.6 | 99.5 |
| **17.9** | 52.6 | 54.3 | 57.2 | 62.6 | 69.7 | 78.4 | 87.9 | 94.6 | 99.5 |
| **18.0** | 52.8 | 54.6 | 57.4 | 62.8 | 69.9 | 78.5 | 88.0 | 94.7 | 99.5 |
| **18.1** | 53.1 | 54.8 | 57.6 | 63.0 | 70.1 | 78.7 | 88.1 | 94.7 | 99.5 |
| **18.2** | 53.3 | 55.0 | 57.8 | 63.2 | 70.3 | 78.8 | 88.2 | 94.7 | 99.5 |
| **18.3** | 53.5 | 55.2 | 58.0 | 63.4 | 70.4 | 78.9 | 88.2 | 94.7 | 99.4 |
| **18.4** | 53.7 | 55.4 | 58.2 | 63.6 | 70.6 | 79.1 | 88.3 | 94.7 | 99.4 |
| **18.5** | 53.8 | 55.6 | 58.4 | 63.8 | 70.8 | 79.2 | 88.3 | 94.7 | 99.3 |
| **18.6** | 54.0 | 55.7 | 58.6 | 63.9 | 70.9 | 79.3 | 88.4 | 94.7 | 99.3 |
| **18.7** | 54.2 | 55.9 | 58.8 | 64.1 | 71.1 | 79.4 | 88.4 | 94.7 | 99.2 |
| **18.8** | 54.4 | 56.1 | 58.9 | 64.3 | 71.2 | 79.5 | 88.5 | 94.7 | 99.2 |
| **18.9** | 54.5 | 56.3 | 59.1 | 64.4 | 71.4 | 79.6 | 88.5 | 94.7 | 99.1 |
| **19.0** | 54.7 | 56.4 | 59.3 | 64.6 | 71.5 | 79.7 | 88.6 | 94.7 | 99.1 |
| **19.1** | 54.8 | 56.6 | 59.4 | 64.7 | 71.6 | 79.8 | 88.6 | 94.7 | 99.0 |
| **19.2** | 55.0 | 56.7 | 59.6 | 64.9 | 71.8 | 79.9 | 88.7 | 94.7 | 98.9 |
| **19.3** | 55.1 | 56.9 | 59.7 | 65.0 | 71.9 | 80.0 | 88.7 | 94.7 | 98.9 |
| **19.4** | 55.3 | 57.0 | 59.9 | 65.2 | 72.0 | 80.1 | 88.7 | 94.7 | 98.8 |
| **19.5** | 55.4 | 57.1 | 60.0 | 65.3 | 72.1 | 80.2 | 88.8 | 94.6 | 98.8 |
| **19.6** | 55.5 | 57.3 | 60.1 | 65.4 | 72.2 | 80.3 | 88.8 | 94.6 | 98.8 |
| **19.7** | 55.7 | 57.4 | 60.2 | 65.5 | 72.3 | 80.3 | 88.8 | 94.6 | 98.7 |
| **19.8** | 55.8 | 57.5 | 60.4 | 65.6 | 72.4 | 80.4 | 88.8 | 94.6 | 98.7 |
| **19.9** | 55.9 | 57.6 | 60.5 | 65.8 | 72.5 | 80.5 | 88.9 | 94.6 | 98.6 |
| **20.0** | 56.0 | 57.7 | 60.6 | 65.9 | 72.6 | 80.5 | 88.9 | 94.6 | 98.6 |

**TABLE E. Women's and men’s BMI**

| **1`l** | **BMI percentiles for women** | | | | | | **BMI percentiles for men** | | | | | |
| --- | --- | --- | --- | --- | --- | --- | --- | --- | --- | --- | --- | --- |
| **Edad** | **P3** | **P5** | **P50** | **85** | **P95** | **P97** | **P3** | **P5** | **P50** | **85** | **P95** | **P97** |
| **3.0** | 13.7 | 13.9 | 15.7 | 17.3 | 18.4 | 18.9 | 13.6 | 13.8 | 15.8 | 17.3 | 18.4 | 18.9 |
| **3.1** | 13.6 | 13.9 | 15.7 | 17.3 | 18.5 | 19.0 | 13.6 | 13.8 | 15.8 | 17.4 | 18.5 | 19.0 |
| **3.2** | 13.6 | 13.9 | 15.7 | 17.4 | 18.6 | 19.1 | 13.6 | 13.8 | 15.8 | 17.4 | 18.6 | 19.1 |
| **3.3** | 13.6 | 13.8 | 15.8 | 17.4 | 18.7 | 19.3 | 13.6 | 13.8 | 15.8 | 17.5 | 18.7 | 19.2 |
| **3.4** | 13.6 | 13.8 | 15.8 | 17.5 | 18.8 | 19.4 | 13.5 | 13.8 | 15.9 | 17.6 | 18.8 | 19.3 |
| **3.5** | 13.6 | 13.8 | 15.8 | 17.6 | 18.9 | 19.5 | 13.5 | 13.8 | 15.9 | 17.6 | 18.9 | 19.4 |
| **3.6** | 13.6 | 13.8 | 15.8 | 17.6 | 19.0 | 19.6 | 13.5 | 13.8 | 15.9 | 17.7 | 19.0 | 19.5 |
| **3.7** | 13.6 | 13.8 | 15.9 | 17.7 | 19.1 | 19.8 | 13.5 | 13.8 | 15.9 | 17.7 | 19.0 | 19.6 |
| **3.8** | 13.5 | 13.8 | 15.9 | 17.8 | 19.2 | 19.9 | 13.5 | 13.8 | 16.0 | 17.8 | 19.1 | 19.7 |
| **3.9** | 13.5 | 13.8 | 15.9 | 17.9 | 19.3 | 20.0 | 13.5 | 13.8 | 16.0 | 17.9 | 19.2 | 19.9 |
| **4.0** | 13.5 | 13.8 | 15.9 | 17.9 | 19.5 | 20.1 | 13.5 | 13.8 | 16.0 | 17.9 | 19.4 | 20.0 |
| **4.1** | 13.5 | 13.8 | 16.0 | 18.0 | 19.6 | 20.3 | 13.5 | 13.8 | 16.0 | 18.0 | 19.5 | 20.1 |
| **4.2** | 13.5 | 13.7 | 16.0 | 18.1 | 19.7 | 20.4 | 13.5 | 13.8 | 16.1 | 18.1 | 19.6 | 20.2 |
| **4.3** | 13.5 | 13.7 | 16.0 | 18.1 | 19.8 | 20.6 | 13.5 | 13.8 | 16.1 | 18.1 | 19.7 | 20.4 |
| **4.4** | 13.5 | 13.7 | 16.0 | 18.2 | 19.9 | 20.7 | 13.5 | 13.8 | 16.1 | 18.2 | 19.8 | 20.5 |
| **4.5** | 13.4 | 13.7 | 16.1 | 18.3 | 20.0 | 20.8 | 13.5 | 13.8 | 16.1 | 18.3 | 19.9 | 20.6 |
| **4.6** | 13.4 | 13.7 | 16.1 | 18.4 | 20.2 | 21.0 | 13.5 | 13.8 | 16.2 | 18.3 | 20.0 | 20.8 |
| **4.7** | 13.4 | 13.7 | 16.1 | 18.4 | 20.3 | 21.1 | 13.5 | 13.8 | 16.2 | 18.4 | 20.1 | 20.9 |
| **4.8** | 13.4 | 13.7 | 16.2 | 18.5 | 20.4 | 21.3 | 13.5 | 13.8 | 16.2 | 18.5 | 20.3 | 21.1 |
| **4.9** | 13.4 | 13.7 | 16.2 | 18.6 | 20.5 | 21.4 | 13.5 | 13.8 | 16.3 | 18.6 | 20.4 | 21.2 |
| **5.0** | 13.4 | 13.7 | 16.2 | 18.7 | 20.7 | 21.6 | 13.5 | 13.8 | 16.3 | 18.6 | 20.5 | 21.4 |
| **5.1** | 13.4 | 13.7 | 16.3 | 18.7 | 20.8 | 21.8 | 13.5 | 13.8 | 16.3 | 18.7 | 20.6 | 21.5 |
| **5.2** | 13.4 | 13.7 | 16.3 | 18.8 | 20.9 | 21.9 | 13.5 | 13.8 | 16.4 | 18.8 | 20.8 | 21.7 |
| **5.3** | 13.4 | 13.7 | 16.3 | 18.9 | 21.0 | 22.1 | 13.5 | 13.8 | 16.4 | 18.9 | 20.9 | 21.9 |
| **5.4** | 13.4 | 13.7 | 16.4 | 19.0 | 21.2 | 22.2 | 13.5 | 13.8 | 16.4 | 18.9 | 21.0 | 22.0 |
| **5.5** | 13.4 | 13.7 | 16.4 | 19.1 | 21.3 | 22.4 | 13.5 | 13.8 | 16.5 | 19.0 | 21.2 | 22.2 |
| **5.6** | 13.4 | 13.7 | 16.4 | 19.2 | 21.5 | 22.6 | 13.5 | 13.8 | 16.5 | 19.1 | 21.3 | 22.4 |
| **5.7** | 13.4 | 13.7 | 16.5 | 19.3 | 21.6 | 22.7 | 13.5 | 13.8 | 16.5 | 19.2 | 21.5 | 22.6 |
| **5.8** | 13.3 | 13.7 | 16.5 | 19.3 | 21.7 | 22.9 | 13.6 | 13.8 | 16.6 | 19.3 | 21.6 | 22.7 |
| **5.9** | 13.3 | 13.7 | 16.5 | 19.4 | 21.9 | 23.1 | 13.6 | 13.9 | 16.6 | 19.4 | 21.8 | 22.9 |
| **6.0** | 13.3 | 13.7 | 16.6 | 19.5 | 22.0 | 23.2 | 13.6 | 13.9 | 16.6 | 19.5 | 21.9 | 23.1 |
| **6.1** | 13.3 | 13.7 | 16.6 | 19.6 | 22.2 | 23.4 | 13.6 | 13.9 | 16.7 | 19.6 | 22.1 | 23.3 |
| **6.2** | 13.3 | 13.7 | 16.7 | 19.7 | 22.3 | 23.6 | 13.6 | 13.9 | 16.7 | 19.6 | 22.2 | 23.5 |
| **6.3** | 13.3 | 13.7 | 16.7 | 19.8 | 22.4 | 23.7 | 13.6 | 13.9 | 16.8 | 19.7 | 22.4 | 23.7 |
| **6.4** | 13.3 | 13.7 | 16.7 | 19.9 | 22.6 | 23.9 | 13.6 | 13.9 | 16.8 | 19.8 | 22.5 | 23.9 |
| **6.5** | 13.3 | 13.7 | 16.8 | 20.0 | 22.7 | 24.1 | 13.6 | 13.9 | 16.8 | 19.9 | 22.7 | 24.1 |
| **6.6** | 13.3 | 13.7 | 16.8 | 20.1 | 22.9 | 24.2 | 13.6 | 13.9 | 16.9 | 20.0 | 22.9 | 24.3 |
| **6.7** | 13.3 | 13.7 | 16.9 | 20.2 | 23.0 | 24.4 | 13.6 | 13.9 | 16.9 | 20.1 | 23.0 | 24.5 |
| **6.8** | 13.4 | 13.7 | 16.9 | 20.3 | 23.2 | 24.6 | 13.6 | 13.9 | 17.0 | 20.2 | 23.2 | 24.7 |
| **6.9** | 13.4 | 13.7 | 17.0 | 20.4 | 23.3 | 24.7 | 13.6 | 14.0 | 17.0 | 20.3 | 23.4 | 24.9 |
| **7.0** | 13.4 | 13.7 | 17.0 | 20.5 | 23.4 | 24.9 | 13.6 | 14.0 | 17.1 | 20.4 | 23.5 | 25.1 |
| **7.1** | 13.4 | 13.7 | 17.1 | 20.5 | 23.6 | 25.1 | 13.7 | 14.0 | 17.1 | 20.5 | 23.7 | 25.3 |
| **7.2** | 13.4 | 13.7 | 17.1 | 20.6 | 23.7 | 25.2 | 13.7 | 14.0 | 17.2 | 20.6 | 23.8 | 25.5 |
| **7.3** | 13.4 | 13.7 | 17.2 | 20.7 | 23.9 | 25.4 | 13.7 | 14.0 | 17.2 | 20.7 | 24.0 | 25.8 |
| **7.4** | 13.4 | 13.8 | 17.2 | 20.8 | 24.0 | 25.6 | 13.7 | 14.0 | 17.3 | 20.8 | 24.2 | 26.0 |
| **7.5** | 13.4 | 13.8 | 17.3 | 20.9 | 24.1 | 25.7 | 13.7 | 14.0 | 17.3 | 20.9 | 24.3 | 26.2 |
| **7.6** | 13.4 | 13.8 | 17.3 | 21.0 | 24.3 | 25.9 | 13.7 | 14.0 | 17.4 | 21.0 | 24.5 | 26.4 |
| **7.7** | 13.4 | 13.8 | 17.4 | 21.1 | 24.4 | 26.0 | 13.7 | 14.1 | 17.4 | 21.1 | 24.7 | 26.6 |
| **7.8** | 13.4 | 13.8 | 17.5 | 21.2 | 24.5 | 26.2 | 13.7 | 14.1 | 17.5 | 21.2 | 24.8 | 26.7 |
| **7.9** | 13.5 | 13.8 | 17.5 | 21.3 | 24.7 | 26.3 | 13.7 | 14.1 | 17.5 | 21.3 | 25.0 | 26.9 |
| **8.0** | 13.5 | 13.9 | 17.6 | 21.4 | 24.8 | 26.5 | 13.8 | 14.1 | 17.6 | 21.4 | 25.1 | 27.1 |
| **8.1** | 13.5 | 13.9 | 17.6 | 21.5 | 24.9 | 26.6 | 13.8 | 14.1 | 17.6 | 21.5 | 25.3 | 27.3 |
| **8.2** | 13.5 | 13.9 | 17.7 | 21.6 | 25.1 | 26.8 | 13.8 | 14.1 | 17.7 | 21.6 | 25.4 | 27.5 |
| **8.3** | 13.5 | 13.9 | 17.8 | 21.7 | 25.2 | 26.9 | 13.8 | 14.2 | 17.7 | 21.7 | 25.6 | 27.7 |
| **8.4** | 13.5 | 14.0 | 17.8 | 21.8 | 25.3 | 27.0 | 13.8 | 14.2 | 17.8 | 21.8 | 25.7 | 27.8 |
| **8.5** | 13.6 | 14.0 | 17.9 | 21.9 | 25.4 | 27.2 | 13.8 | 14.2 | 17.9 | 21.9 | 25.9 | 28.0 |
| **8.6** | 13.6 | 14.0 | 17.9 | 22.0 | 25.6 | 27.3 | 13.9 | 14.2 | 17.9 | 22.0 | 26.0 | 28.2 |
| **8.7** | 13.6 | 14.0 | 18.0 | 22.1 | 25.7 | 27.4 | 13.9 | 14.2 | 18.0 | 22.2 | 26.2 | 28.3 |
| **8.8** | 13.6 | 14.1 | 18.1 | 22.2 | 25.8 | 27.6 | 13.9 | 14.3 | 18.0 | 22.3 | 26.3 | 28.5 |
| **8.9** | 13.7 | 14.1 | 18.1 | 22.3 | 25.9 | 27.7 | 13.9 | 14.3 | 18.1 | 22.4 | 26.5 | 28.6 |
| **9.0** | 13.7 | 14.1 | 18.2 | 22.4 | 26.1 | 27.8 | 13.9 | 14.3 | 18.1 | 22.4 | 26.6 | 28.8 |
| **9.1** | 13.7 | 14.1 | 18.3 | 22.5 | 26.2 | 28.0 | 13.9 | 14.3 | 18.2 | 22.5 | 26.7 | 28.9 |
| **9.2** | 13.7 | 14.2 | 18.3 | 22.6 | 26.3 | 28.1 | 14.0 | 14.4 | 18.3 | 22.6 | 26.8 | 29.1 |
| **9.3** | 13.8 | 14.2 | 18.4 | 22.7 | 26.4 | 28.2 | 14.0 | 14.4 | 18.3 | 22.7 | 27.0 | 29.2 |
| **9.4** | 13.8 | 14.2 | 18.5 | 22.8 | 26.5 | 28.3 | 14.0 | 14.4 | 18.4 | 22.8 | 27.1 | 29.3 |
| **9.5** | 13.8 | 14.3 | 18.5 | 22.9 | 26.6 | 28.5 | 14.0 | 14.4 | 18.4 | 22.9 | 27.2 | 29.5 |
| **9.6** | 13.9 | 14.3 | 18.6 | 23.0 | 26.8 | 28.6 | 14.1 | 14.5 | 18.5 | 23.0 | 27.3 | 29.6 |
| **9.7** | 13.9 | 14.3 | 18.7 | 23.1 | 26.9 | 28.7 | 14.1 | 14.5 | 18.6 | 23.1 | 27.4 | 29.7 |
| **9.8** | 13.9 | 14.4 | 18.7 | 23.2 | 27.0 | 28.8 | 14.1 | 14.5 | 18.6 | 23.2 | 27.5 | 29.8 |
| **9.9** | 14.0 | 14.4 | 18.8 | 23.3 | 27.1 | 28.9 | 14.1 | 14.6 | 18.7 | 23.3 | 27.6 | 29.9 |
| **10.0** | 14.0 | 14.5 | 18.9 | 23.4 | 27.2 | 29.0 | 14.2 | 14.6 | 18.8 | 23.4 | 27.7 | 30.0 |
| **10.1** | 14.0 | 14.5 | 18.9 | 23.5 | 27.3 | 29.2 | 14.2 | 14.6 | 18.8 | 23.5 | 27.8 | 30.1 |
| **10.2** | 14.1 | 14.5 | 19.0 | 23.6 | 27.4 | 29.3 | 14.2 | 14.7 | 18.9 | 23.6 | 27.9 | 30.2 |
| **10.3** | 14.1 | 14.6 | 19.1 | 23.7 | 27.5 | 29.4 | 14.2 | 14.7 | 18.9 | 23.6 | 28.0 | 30.3 |
| **10.4** | 14.1 | 14.6 | 19.1 | 23.7 | 27.6 | 29.5 | 14.3 | 14.7 | 19.0 | 23.7 | 28.1 | 30.4 |
| **10.5** | 14.2 | 14.7 | 19.2 | 23.8 | 27.7 | 29.6 | 14.3 | 14.7 | 19.1 | 23.8 | 28.2 | 30.5 |
| **10.6** | 14.2 | 14.7 | 19.3 | 23.9 | 27.8 | 29.7 | 14.3 | 14.8 | 19.1 | 23.9 | 28.3 | 30.5 |
| **10.7** | 14.3 | 14.7 | 19.4 | 24.0 | 27.9 | 29.8 | 14.4 | 14.8 | 19.2 | 24.0 | 28.3 | 30.6 |
| **10.8** | 14.3 | 14.8 | 19.4 | 24.1 | 28.0 | 29.9 | 14.4 | 14.9 | 19.2 | 24.0 | 28.4 | 30.7 |
| **10.9** | 14.3 | 14.8 | 19.5 | 24.2 | 28.1 | 30.0 | 14.4 | 14.9 | 19.3 | 24.1 | 28.5 | 30.7 |
| **11.0** | 14.4 | 14.9 | 19.6 | 24.3 | 28.2 | 30.1 | 14.5 | 14.9 | 19.4 | 24.2 | 28.6 | 30.8 |
| **11.1** | 14.4 | 14.9 | 19.6 | 24.3 | 28.3 | 30.2 | 14.5 | 15.0 | 19.4 | 24.3 | 28.6 | 30.9 |
| **11.2** | 14.5 | 15.0 | 19.7 | 24.4 | 28.4 | 30.2 | 14.5 | 15.0 | 19.5 | 24.3 | 28.7 | 30.9 |
| **11.3** | 14.5 | 15.0 | 19.8 | 24.5 | 28.5 | 30.3 | 14.6 | 15.0 | 19.5 | 24.4 | 28.8 | 31.0 |
| **11.4** | 14.6 | 15.1 | 19.8 | 24.6 | 28.5 | 30.4 | 14.6 | 15.1 | 19.6 | 24.5 | 28.8 | 31.0 |
| **11.5** | 14.6 | 15.1 | 19.9 | 24.7 | 28.6 | 30.5 | 14.7 | 15.1 | 19.7 | 24.5 | 28.9 | 31.1 |
| **11.6** | 14.7 | 15.2 | 19.9 | 24.7 | 28.7 | 30.6 | 14.7 | 15.2 | 19.7 | 24.6 | 29.0 | 31.1 |
| **11.7** | 14.7 | 15.2 | 20.0 | 24.8 | 28.8 | 30.6 | 14.7 | 15.2 | 19.8 | 24.7 | 29.0 | 31.2 |
| **11.8** | 14.8 | 15.3 | 20.1 | 24.9 | 28.8 | 30.7 | 14.8 | 15.3 | 19.8 | 24.7 | 29.1 | 31.2 |
| **11.9** | 14.8 | 15.3 | 20.1 | 24.9 | 28.9 | 30.8 | 14.8 | 15.3 | 19.9 | 24.8 | 29.1 | 31.3 |
| **12.0** | 14.9 | 15.4 | 20.2 | 25.0 | 29.0 | 30.8 | 14.9 | 15.4 | 20.0 | 24.9 | 29.2 | 31.3 |
| **12.1** | 14.9 | 15.4 | 20.3 | 25.1 | 29.0 | 30.9 | 14.9 | 15.4 | 20.0 | 24.9 | 29.2 | 31.4 |
| **12.2** | 15.0 | 15.5 | 20.3 | 25.1 | 29.1 | 31.0 | 15.0 | 15.5 | 20.1 | 25.0 | 29.3 | 31.4 |
| **12.3** | 15.0 | 15.5 | 20.4 | 25.2 | 29.2 | 31.0 | 15.0 | 15.5 | 20.1 | 25.0 | 29.3 | 31.5 |
| **12.4** | 15.1 | 15.6 | 20.4 | 25.3 | 29.2 | 31.1 | 15.1 | 15.6 | 20.2 | 25.1 | 29.4 | 31.5 |
| **12.5** | 15.1 | 15.6 | 20.5 | 25.3 | 29.3 | 31.1 | 15.1 | 15.6 | 20.3 | 25.2 | 29.4 | 31.6 |
| **12.6** | 15.2 | 15.7 | 20.6 | 25.4 | 29.3 | 31.2 | 15.2 | 15.7 | 20.3 | 25.2 | 29.5 | 31.6 |
| **12.7** | 15.2 | 15.7 | 20.6 | 25.4 | 29.4 | 31.2 | 15.2 | 15.7 | 20.4 | 25.3 | 29.5 | 31.6 |
| **12.8** | 15.3 | 15.8 | 20.7 | 25.5 | 29.4 | 31.3 | 15.3 | 15.8 | 20.4 | 25.3 | 29.6 | 31.7 |
| **12.9** | 15.3 | 15.8 | 20.7 | 25.5 | 29.5 | 31.3 | 15.3 | 15.8 | 20.5 | 25.4 | 29.6 | 31.7 |
| **13.0** | 15.4 | 15.9 | 20.8 | 25.6 | 29.5 | 31.3 | 15.4 | 15.9 | 20.6 | 25.4 | 29.7 | 31.8 |
| **13.1** | 15.4 | 16.0 | 20.8 | 25.6 | 29.5 | 31.4 | 15.4 | 15.9 | 20.6 | 25.5 | 29.7 | 31.8 |
| **13.2** | 15.5 | 16.0 | 20.9 | 25.7 | 29.6 | 31.4 | 15.5 | 16.0 | 20.7 | 25.5 | 29.8 | 31.8 |
| **13.3** | 15.5 | 16.1 | 20.9 | 25.7 | 29.6 | 31.4 | 15.5 | 16.0 | 20.7 | 25.6 | 29.8 | 31.9 |
| **13.4** | 15.6 | 16.1 | 21.0 | 25.7 | 29.6 | 31.4 | 15.6 | 16.1 | 20.8 | 25.6 | 29.9 | 31.9 |
| **13.5** | 15.7 | 16.2 | 21.0 | 25.8 | 29.6 | 31.5 | 15.6 | 16.1 | 20.8 | 25.7 | 29.9 | 32.0 |
| **13.6** | 15.7 | 16.2 | 21.1 | 25.8 | 29.7 | 31.5 | 15.7 | 16.2 | 20.9 | 25.8 | 29.9 | 32.0 |
| **13.7** | 15.8 | 16.3 | 21.1 | 25.8 | 29.7 | 31.5 | 15.8 | 16.3 | 21.0 | 25.8 | 30.0 | 32.0 |
| **13.8** | 15.8 | 16.3 | 21.1 | 25.8 | 29.7 | 31.5 | 15.8 | 16.3 | 21.0 | 25.9 | 30.0 | 32.1 |
| **13.9** | 15.9 | 16.4 | 21.2 | 25.9 | 29.7 | 31.5 | 15.9 | 16.4 | 21.1 | 25.9 | 30.1 | 32.1 |
| **14.0** | 15.9 | 16.5 | 21.2 | 25.9 | 29.7 | 31.5 | 15.9 | 16.4 | 21.1 | 26.0 | 30.1 | 32.1 |
| **14.1** | 16.0 | 16.5 | 21.3 | 25.9 | 29.7 | 31.5 | 16.0 | 16.5 | 21.2 | 26.0 | 30.1 | 32.2 |
| **14.2** | 16.0 | 16.6 | 21.3 | 25.9 | 29.7 | 31.5 | 16.0 | 16.5 | 21.2 | 26.0 | 30.2 | 32.2 |
| **14.3** | 16.1 | 16.6 | 21.3 | 25.9 | 29.7 | 31.5 | 16.1 | 16.6 | 21.3 | 26.1 | 30.2 | 32.2 |
| **14.4** | 16.2 | 16.7 | 21.4 | 26.0 | 29.7 | 31.5 | 16.2 | 16.7 | 21.3 | 26.1 | 30.3 | 32.3 |
| **14.5** | 16.2 | 16.7 | 21.4 | 26.0 | 29.7 | 31.5 | 16.2 | 16.7 | 21.4 | 26.2 | 30.3 | 32.3 |
| **14.6** | 16.3 | 16.8 | 21.4 | 26.0 | 29.7 | 31.4 | 16.3 | 16.8 | 21.4 | 26.2 | 30.3 | 32.3 |
| **14.7** | 16.3 | 16.8 | 21.4 | 26.0 | 29.7 | 31.4 | 16.3 | 16.8 | 21.5 | 26.3 | 30.4 | 32.3 |
| **14.8** | 16.4 | 16.9 | 21.5 | 26.0 | 29.7 | 31.4 | 16.4 | 16.9 | 21.6 | 26.3 | 30.4 | 32.4 |
| **14.9** | 16.4 | 16.9 | 21.5 | 26.0 | 29.6 | 31.4 | 16.5 | 17.0 | 21.6 | 26.4 | 30.4 | 32.4 |
| **15.0** | 16.5 | 17.0 | 21.5 | 26.0 | 29.6 | 31.3 | 16.5 | 17.0 | 21.7 | 26.4 | 30.5 | 32.4 |
| **15.1** | 16.5 | 17.0 | 21.5 | 26.0 | 29.6 | 31.3 | 16.6 | 17.1 | 21.7 | 26.4 | 30.5 | 32.4 |
| **15.2** | 16.6 | 17.1 | 21.5 | 26.0 | 29.6 | 31.3 | 16.6 | 17.1 | 21.8 | 26.5 | 30.5 | 32.5 |
| **15.3** | 16.6 | 17.1 | 21.6 | 25.9 | 29.5 | 31.2 | 16.7 | 17.2 | 21.8 | 26.5 | 30.5 | 32.5 |
| **15.4** | 16.7 | 17.1 | 21.6 | 25.9 | 29.5 | 31.2 | 16.7 | 17.2 | 21.9 | 26.5 | 30.5 | 32.5 |
| **15.5** | 16.7 | 17.2 | 21.6 | 25.9 | 29.5 | 31.1 | 16.8 | 17.3 | 21.9 | 26.6 | 30.6 | 32.5 |
| **15.6** | 16.8 | 17.2 | 21.6 | 25.9 | 29.4 | 31.1 | 16.9 | 17.4 | 21.9 | 26.6 | 30.6 | 32.5 |
| **15.7** | 16.8 | 17.3 | 21.6 | 25.9 | 29.4 | 31.0 | 16.9 | 17.4 | 22.0 | 26.6 | 30.6 | 32.5 |
| **15.8** | 16.8 | 17.3 | 21.6 | 25.9 | 29.3 | 31.0 | 17.0 | 17.5 | 22.0 | 26.7 | 30.6 | 32.5 |
| **15.9** | 16.9 | 17.3 | 21.6 | 25.8 | 29.3 | 30.9 | 17.0 | 17.5 | 22.1 | 26.7 | 30.6 | 32.5 |
| **16.0** | 16.9 | 17.4 | 21.6 | 25.8 | 29.2 | 30.9 | 17.1 | 17.6 | 22.1 | 26.7 | 30.6 | 32.5 |
| **16.1** | 17.0 | 17.4 | 21.7 | 25.8 | 29.2 | 30.8 | 17.1 | 17.6 | 22.2 | 26.8 | 30.7 | 32.5 |
| **16.2** | 17.0 | 17.5 | 21.7 | 25.8 | 29.1 | 30.7 | 17.2 | 17.7 | 22.2 | 26.8 | 30.7 | 32.5 |
| **16.3** | 17.0 | 17.5 | 21.7 | 25.7 | 29.1 | 30.7 | 17.2 | 17.7 | 22.3 | 26.8 | 30.7 | 32.5 |
| **16.4** | 17.1 | 17.5 | 21.7 | 25.7 | 29.0 | 30.6 | 17.3 | 17.8 | 22.3 | 26.8 | 30.7 | 32.5 |
| **16.5** | 17.1 | 17.5 | 21.7 | 25.7 | 29.0 | 30.5 | 17.3 | 17.8 | 22.3 | 26.9 | 30.7 | 32.5 |
| **16.6** | 17.1 | 17.6 | 21.7 | 25.7 | 28.9 | 30.5 | 17.4 | 17.9 | 22.4 | 26.9 | 30.7 | 32.5 |
| **16.7** | 17.2 | 17.6 | 21.7 | 25.6 | 28.9 | 30.4 | 17.4 | 17.9 | 22.4 | 26.9 | 30.7 | 32.5 |
| **16.8** | 17.2 | 17.6 | 21.7 | 25.6 | 28.8 | 30.3 | 17.5 | 18.0 | 22.4 | 26.9 | 30.7 | 32.5 |
| **16.9** | 17.2 | 17.7 | 21.7 | 25.6 | 28.8 | 30.3 | 17.5 | 18.0 | 22.5 | 27.0 | 30.7 | 32.5 |
| **17.0** | 17.2 | 17.7 | 21.7 | 25.6 | 28.7 | 30.2 | 17.6 | 18.0 | 22.5 | 27.0 | 30.7 | 32.5 |
| **17.1** | 17.3 | 17.7 | 21.7 | 25.5 | 28.7 | 30.1 | 17.6 | 18.1 | 22.6 | 27.0 | 30.7 | 32.5 |
| **17.2** | 17.3 | 17.7 | 21.7 | 25.5 | 28.6 | 30.1 | 17.7 | 18.1 | 22.6 | 27.0 | 30.7 | 32.4 |
| **17.3** | 17.3 | 17.7 | 21.7 | 25.5 | 28.5 | 30.0 | 17.7 | 18.2 | 22.6 | 27.0 | 30.7 | 32.4 |
| **17.4** | 17.3 | 17.8 | 21.7 | 25.4 | 28.5 | 29.9 | 17.7 | 18.2 | 22.7 | 27.0 | 30.7 | 32.4 |
| **17.5** | 17.4 | 17.8 | 21.7 | 25.4 | 28.4 | 29.8 | 17.8 | 18.3 | 22.7 | 27.1 | 30.7 | 32.4 |
| **17.6** | 17.4 | 17.8 | 21.7 | 25.4 | 28.4 | 29.8 | 17.8 | 18.3 | 22.7 | 27.1 | 30.7 | 32.3 |
| **17.7** | 17.4 | 17.8 | 21.7 | 25.3 | 28.3 | 29.7 | 17.9 | 18.4 | 22.8 | 27.1 | 30.6 | 32.3 |
| **17.8** | 17.4 | 17.8 | 21.6 | 25.3 | 28.2 | 29.6 | 17.9 | 18.4 | 22.8 | 27.1 | 30.6 | 32.3 |
| **17.9** | 17.4 | 17.8 | 21.6 | 25.3 | 28.2 | 29.5 | 18.0 | 18.4 | 22.8 | 27.1 | 30.6 | 32.3 |
| **18.0** | 17.4 | 17.9 | 21.6 | 25.2 | 28.1 | 29.5 | 18.0 | 18.5 | 22.9 | 27.1 | 30.6 | 32.2 |
| **18.1** | 17.5 | 17.9 | 21.6 | 25.2 | 28.1 | 29.4 | 18.0 | 18.5 | 22.9 | 27.1 | 30.6 | 32.2 |
| **18.2** | 17.5 | 17.9 | 21.6 | 25.2 | 28.0 | 29.3 | 18.1 | 18.6 | 22.9 | 27.2 | 30.6 | 32.2 |
| **18.3** | 17.5 | 17.9 | 21.6 | 25.1 | 27.9 | 29.3 | 18.1 | 18.6 | 23.0 | 27.2 | 30.5 | 32.1 |
| **18.4** | 17.5 | 17.9 | 21.6 | 25.1 | 27.9 | 29.2 | 18.1 | 18.6 | 23.0 | 27.2 | 30.5 | 32.1 |
| **18.5** | 17.5 | 17.9 | 21.6 | 25.1 | 27.8 | 29.1 | 18.2 | 18.7 | 23.0 | 27.2 | 30.5 | 32.1 |
| **18.6** | 17.5 | 17.9 | 21.6 | 25.0 | 27.8 | 29.1 | 18.2 | 18.7 | 23.0 | 27.2 | 30.5 | 32.0 |
| **18.7** | 17.5 | 17.9 | 21.6 | 25.0 | 27.7 | 29.0 | 18.3 | 18.7 | 23.1 | 27.2 | 30.5 | 32.0 |
| **18.8** | 17.5 | 17.9 | 21.6 | 25.0 | 27.7 | 28.9 | 18.3 | 18.8 | 23.1 | 27.2 | 30.4 | 31.9 |
| **18.9** | 17.5 | 17.9 | 21.6 | 24.9 | 27.6 | 28.9 | 18.3 | 18.8 | 23.1 | 27.2 | 30.4 | 31.9 |
| **19.0** | 17.5 | 17.9 | 21.5 | 24.9 | 27.6 | 28.8 | 18.4 | 18.8 | 23.2 | 27.2 | 30.4 | 31.9 |
| **19.1** | 17.6 | 18.0 | 21.5 | 24.9 | 27.5 | 28.7 | 18.4 | 18.9 | 23.2 | 27.2 | 30.4 | 31.8 |
| **19.2** | 17.6 | 18.0 | 21.5 | 24.9 | 27.5 | 28.7 | 18.4 | 18.9 | 23.2 | 27.2 | 30.3 | 31.8 |
| **19.3** | 17.6 | 18.0 | 21.5 | 24.8 | 27.4 | 28.6 | 18.5 | 18.9 | 23.2 | 27.2 | 30.3 | 31.7 |
| **19.4** | 17.6 | 18.0 | 21.5 | 24.8 | 27.4 | 28.6 | 18.5 | 19.0 | 23.3 | 27.2 | 30.3 | 31.7 |
| **19.5** | 17.6 | 18.0 | 21.5 | 24.8 | 27.3 | 28.5 | 18.5 | 19.0 | 23.3 | 27.2 | 30.3 | 31.7 |
| **19.6** | 17.6 | 18.0 | 21.5 | 24.7 | 27.3 | 28.5 | 18.6 | 19.0 | 23.3 | 27.2 | 30.2 | 31.6 |
| **19.7** | 17.6 | 18.0 | 21.5 | 24.7 | 27.3 | 28.4 | 18.6 | 19.1 | 23.3 | 27.2 | 30.2 | 31.6 |
| **19.8** | 17.6 | 18.0 | 21.5 | 24.7 | 27.2 | 28.4 | 18.6 | 19.1 | 23.4 | 27.2 | 30.2 | 31.5 |
| **19.9** | 17.6 | 18.0 | 21.5 | 24.7 | 27.2 | 28.3 | 18.7 | 19.1 | 23.4 | 27.2 | 30.2 | 31.5 |
| **20.0** | 17.6 | 18.0 | 21.4 | 24.6 | 27.1 | 28.3 | 18.7 | 19.2 | 23.4 | 27.2 | 30.2 | 31.5 |
